# Pulmonary Vascular Endothelial Cells in Lung Diseases: Mechanisms, Therapeutic Strategies, and Future Directions

**DOI:** 10.1111/cpr.70136

**Published:** 2025-10-15

**Authors:** Qianyue Liu, Hongshuai Zheng, Jing Liu, Ming Gao, Faquan Lin, Lin Liao

**Affiliations:** ^1^ Department of Clinical Laboratory The First Affiliated Hospital of Guangxi Medical University Nanning China; ^2^ Key Laboratory of Clinical Laboratory Medicine of Guangxi Medical University Education Department of Guangxi Zhuang Autonomous Region Nanning China; ^3^ Life Sciences Institute Guangxi Medical University Nanning China

**Keywords:** anti‐aging therapy, genetic therapy, immunomodulation, metabolic homeostasis, pulmonary diseases, vascular endothelial cells

## Abstract

Pulmonary vascular endothelial cells (VECs) are essential for the normal function of the lung, through maintaining vascular barrier integrity, regulating blood flow, and participating in inflammatory responses to safeguard oxygen exchange and physiological homeostasis. The occurrence and development of various pulmonary diseases all take the injury of pulmonary VECs as an important pathological hub, which directly affects the therapeutic effect and prognosis recovery of patients. The injury mechanisms of pulmonary VECs present multi‐dimensional network characteristics, involving inflammation and oxidative stress, genetic factors, cellular senescence, metabolic abnormalities, and immune dysregulation. Due to the unique physiological structure of the lungs, traditional drugs often encounter significant challenges in clinical application such as insufficient targeting, low bioavailability, and systemic side effects. In order to overcome the existing treatment bottlenecks, it is crucial to implement an in‐depth analysis of the molecular mechanism of pulmonary VECs injury. This review systematically explores the mechanisms of pulmonary VECs injury, evaluates novel therapeutic strategies targeting pulmonary VECs' dysfunction, and discusses the challenges and future prospects of clinical translation. The goal is to shift pulmonary diseases treatment from symptom management to precise molecular intervention.

## Graphical abstract

The pathogenic mechanisms of pulmonary vascular endothelial cells (VECs) in lung diseases and their multimodal therapeutic strategies.

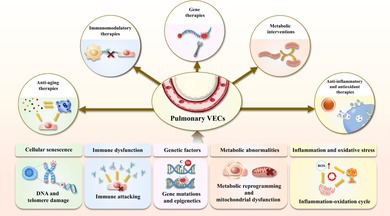

## 1 Introduction

Acute and chronic pulmonary diseases remain a highly challenging issue globally. Statistical data indicate that the number of deaths from chronic obstructive pulmonary disease (COPD) is on a rising trend worldwide, with notable increases in western countries. Specifically, the mortality rate of COPD has surged by 5% in the United Kingdom and 10% in the United States [1]. Despite the advancements in the aetiology and treatment of these diseases, their underlying mechanisms and long‐term efficacy remain elusive. Although earlier research primarily concentrated on the damage and degeneration of alveolar epithelial cells, the emerging evidence highlighted the crucial role of pulmonary vascular endothelial cells (VECs) in pulmonary diseases [2, 3].

Pulmonary VECs are a monolayer of flat epithelial cells on the inner surface of pulmonary blood vessels. They form a dynamic barrier through the connection of tight junction proteins, which preserve vascular integrity and prevent fluid and protein leakage into alveolar spaces. This barrier is crucial for the alveolar‐capillary interface, facilitating the efficient diffusion of oxygen and carbon dioxide [4]. They also play crucial roles in regulating vascular tone, immune and inflammatory responses, coagulation balance, and metabolic and endocrine functions, thereby maintaining pulmonary circulation homeostasis. The disruption of these functions can trigger severe reactions, including hypoxemia, cardiac dysfunction, hypercapnia, and liver function impairment [5]. The impact of pulmonary VECs is not limited to pulmonary diseases; it also seriously damages the quality of life and physical health of patients.

Current treatments targeting pulmonary VECs primarily rely on drug therapy, including anti‐inflammatory agents, antioxidants, antithrombotic drugs, and vasodilators [6, 7, 8, 9, 10, 11, 12, 13]. For example, glucocorticoids (GCs) are the predominant anti‐inflammatory drugs used clinically and offer robust protection against pulmonary VECs damage [14]. However, long‐term use can result in immunosuppression and metabolic disorders [15]. Additionally, the first‐pass effect significantly diminishes the drug's bioavailability, leading to suboptimal concentrations reaching the pulmonary VECs [16]. Although the pulmonary VECs have a large surface area, offering numerous opportunities for drug exposure, rapid blood flow limits the interaction time between drugs and endothelium. Although the loose connections between pulmonary VECs facilitate the penetration of small molecules, large or polar drugs may face limitations. Pathological conditions can lead to endothelial cell activation, barrier dysfunction, and altered drug uptake, further affecting drug efficacy. The current challenges, including drug side effects, inadequate targeting, and limited efficacy in treating lung diseases, highlight the need for strategies that precisely target pulmonary VECs [17, 18]. As the central regulatory element in lung disease progression, pulmonary VECs offer a biological basis for targeted intervention. By developing therapies that specifically target pulmonary VECs, it can enhance drug targeting and bioavailability, minimise systemic side effects, and interrupt pathological processes at an early stage, thereby significantly improving therapeutic outcomes.

This review systematically elucidates the multidimensional pathogenic mechanisms of pulmonary VECs in lung diseases, including inflammation, oxidative stress, genetic factors, cellular senescence, abnormal cellular metabolism, and immune dysfunction, and provides a theoretical basis for therapeutic strategies targeting pulmonary VECs. On this basis, innovative therapeutic strategies targeting pulmonary VECs dysfunction are further explored, and challenges to their clinical translation are dissected. This review aims to bridge basic research and clinical application, provide new insights into the central role of pulmonary VECs in lung diseases, emphasise the core value of targeting pulmonary VECs in overcoming the current therapeutic dilemma, and advocate a shift in the treatment of lung diseases from symptom management to molecular mechanism intervention.

## 2 Pathogenesis of Pulmonary VECs

**FIGURE 1:**
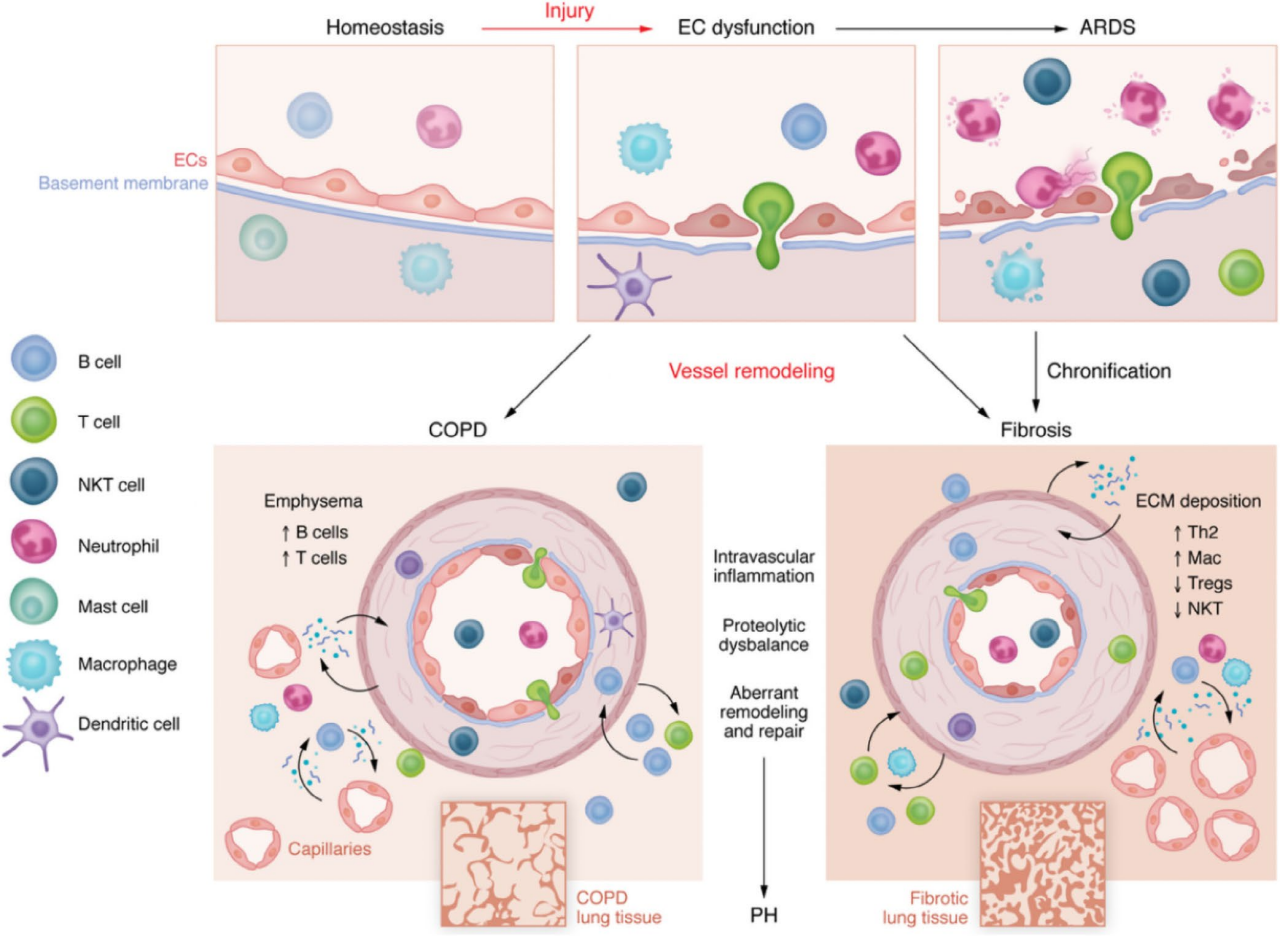
Pathological changes of pulmonary VECs in lung diseases. Harmful factors damage pulmonary EC, leading to the disruption of vascular homeostasis. Activate EC and cause the loss of its barrier function, thereby promoting white blood cell adhesion and transendothelial migration. Aggravated EC dysfunction, intensified inflammatory responses and the recruitment of white blood cells can lead to an inflammatory storm, which in turn causes tissue damage and subsequent remodelling. If not resolved, or if the persistent tissue damage is too severe, this process may lead to the onset of progressive fibrosis in the lung tissue. ARDS, acute respiratory distress syndrome; COPD, chronic obstructive pulmonary disease; EC, endothelial cell; PF, pulmonary fibrosis; PH, pulmonary hypertension. Reprinted from Borek et al., 2023 with permission [2].

Pulmonary VECs are the core hub of the occurrence and development of various lung diseases, and they are a key driver for the progression of these diseases [2, 19, 20, 21, 22]. In acute lung‐related diseases such as acute lung injury (ALI) and acute respiratory distress syndrome (ARDS), the continuous attack of pathogens and inflammatory factors will directly cause the destruction of the structural integrity of pulmonary VECs, specifically manifested as the loosening of intercellular connections and the interruption of continuity [2, 20]. This structural damage leads to the failure of the barrier function of pulmonary VECs, thereby triggering a severe inflammatory response. The pathological changes not only cause the leakage of protein‐rich fluid and the formation of alveolar cavity pulmonary edema but also severely hamper the gas exchange function by alveolar‐capillary membrane thickening, eventually leading to acute lung dysfunction [23]. If the pulmonary VECs' injury state persists, their phenotype will undergo significant transformation from a normal anticoagulant and anti‐inflammatory phenotype to a procoagulant and pro‐inflammatory phenotype, and secrete fibrosis‐related factors to promote the process of pulmonary fibrosis (PF) [22, 24]. When PF occurs, the normal structure of the lung is replaced by a large number of collagen fibres, which leads to changes in pulmonary hemodynamics and causes local pulmonary VECs to be in a hypoxic environment. This hypoxia can induce changes in the function of pulmonary VECs, promote pulmonary artery constriction and intimal hyperplasia, and ultimately lead to pulmonary hypertension (PH) [19]. In addition, chronic inflammation, caused by toxins, smoke and so forth, will accelerate the aging process of pulmonary VECs in COPD [21]. It leads to a decrease in the density of the capillary network, intensifies the changes in vascular remodelling, and forms a synergistic deterioration effect of PF and PH. The pathological process centered on pulmonary VECs forms a unique triangular closed loop of ‘leakage ‐ hypoxia – remodelling’ throughout the entire process of lung diseases. Figure 1 shows the entire evolution process of lung diseases centered on pulmonary VECs.

### 2.1 Inflammation and Oxidative Stress

Pulmonary VECs are central targets and effector cells of inflammation and oxidative stress in the lung. Activated pulmonary VECs respond to damage associated molecular patterns (DAMPs) or pathogen associated molecular patterns (PAMPs) through pattern recognition receptors (PRRs). It triggers the nuclear factor‐κB (NF‐κB) signalling pathway, thereby enhancing the secretion of inflammatory factors such as interleukin‐6 (IL‐6) and interleukin‐8 (IL‐8) [25, 26]. The mitogen activated protein kinase (MAPK) pathway acts synergistically in this process, where phosphorylation of p38 induces the expression of tumour necrosis factor‐α (TNF‐α) and interleukin‐1β (IL‐1β) through the regulation of inflammatory gene transcription and mRNA stability [27]. In addition, the activation of nucleotide binding oligomerised structural domain like receptor family pyrin domain containing protein 3 (NLRP3) inflammasome promotes caspase‐1 mediated maturation and release of IL‐1β and IL‐8 [28]. The robust release of inflammatory mediators, and increased vascular permeability collaboratively drive the extensive activation and migration of inflammatory cells, notably neutrophils and monocytes to the site of injury [28, 29]. Besides, the upregulation of adhesion molecules on pulmonary VECs facilitates the rolling and adhesion of inflammatory cells on the surface. Meanwhile, neutrophil extracellular traps (NETs) and various secreted proteases not only respond to noxious stimuli but also intensify pulmonary VECs damage, perpetuating a local inflammatory milieu and establishing a self‐perpetuating cycle of inflammation [30, 31].

Significantly, endothelial interstitial transformation (EndMT) is a characteristic pathological change that occurs when inflammation persists [32]. This process is the core mechanism by which pulmonary VECs mediate the pathological progression of chronic lung diseases. Its continuous activation will gradually promote the irreversible development of PF. The study by Gaikwad et al. on patients with idiopathic pulmonary fibrosis (IPF) quantified the driving factors of EndMT. It was confirmed that the active transforming growth factor‐β (TGF‐β)/Smad‐2/3 dependent pathway and β‐catenin (β‐catenin) dependent Wnt signalling pathway were involved in regulating EndMT in patients with IPF [33]. Meanwhile, TGF‐β1 activated endothelial Smad pathway was also observed in lung tissue sections of COPD patients [34]. In a recent study, a dual homologous recombinase genetic strategy revealed that Plvap^+^ endothelial cells responded to injury in pulmonary arterial hypertension (PAH) with transient activation of the mesenchymal gene α‐SMA, suggesting that a specific endothelial subpopulation might be a major contributor to EndMT [35]. However, there are still significant limitations in current EndMT studies, including the differences in the response of different pulmonary VECs subpopulations to inflammatory signals, the spatial and temporal dynamics of EndMT initiation, and the key molecular networks that mediate the inflammation–EndMT–fibrosis cascade, etc. In the future, it is expected that the combination of single cell multigenomics and spatial transcriptomics will further elucidate the mechanisms of subpopulation specific regulation and the network of cross‐cellular interactions.

Oxidative stress usually occurs in conjunction with inflammatory responses and is characterised by excessive production of reactive oxygen species (ROS) within pulmonary VECs [36]. This phenomenon is primarily attributed to the combined impact of dysregulated activation of endogenous enzymes, disruption of the antioxidant defence system, and endoplasmic reticulum stress (ERS). The nicotinamide adenine dinucleotide phosphate oxidase (NOX) family serves as the primary endogenous enzyme systems accountable for the generation of ROS within pulmonary VECs [37]. Generally, human cells exhibit seven distinct subtypes of NOX, with NOX1, NOX2, NOX4, and NOX5 being prevalent in pulmonary VECs [37, 38]. Under normal circumstances, NOX is constitutively expressed, facilitating the controllable production of ROS at modest levels, which plays a pivotal role as signalling molecules that control vascular function and structure [39]. However, in response to inflammatory stimuli, NOX subtypes are activated via phosphorylation or Rac1 guanosine triphosphate hydrolase (GTPase), facilitating electron transfer from nicotinamide adenine dinucleotide phosphate (NADPH) to oxygen and leading to the formation of superoxide anions [38]. Apart from the inflammatory triggers, external factors like hypoxia, elevated glucose levels, and hypercholesterolemia can also trigger aberrant activation of this pathway. For instance, for patients with PAH, it has been observed that the confluence of hypoxia and shear stress can amplify ROS production in pulmonary VECs through the NOX1 and C‐X‐C motif chemokine ligand 12 (CXCL12) signalling pathway, thereby promoting the proliferation phenotype [40, 41, 42].

Oxidative stress and ERS form a vicious cycle. It has been pointed out that in the mouse model of ALI, lipopolysaccharide (LPS) stimulation triggers the activation of the mammalian target of rapamycin (mTOR) pathway, stimulating the synthesis of inflammation‐related protein [43]. Consequently, there is an accumulation of unfolded or misfolded proteins in the endoplasmic reticulum, leading to ERS [43, 44]. ERS is characterised by the increased levels of ROS and malondialdehyde (MDA), along with reduced superoxide dismutase (SOD) activity, thereby promoting oxidative stress [45]. The oxidative stress exacerbates the disorder of ER protein folding capacity, establishing a detrimental cycle. Significantly, ER is intricately linked to the inflammatory response, facilitating the release of inflammatory mediators from pulmonary VECs through pathways like inositol‐requiring enzyme 1α (IRE1α)‐X‐box binding protein 1 (XBP1) and inflammasome activation [46, 47]. This process can disrupt the expression of pulmonary VECs' tight junction proteins, modulate vascular permeability, and amplify the inflammatory cascade. Besides, the secretion of inflammatory factors such as TNF‐α can disrupt the calcium homeostasis of ER, leading to the accumulation of unfolded proteins, activating the unfolded protein response (UPR), and exacerbating ERS [47, 48].

**FIGURE 2:**
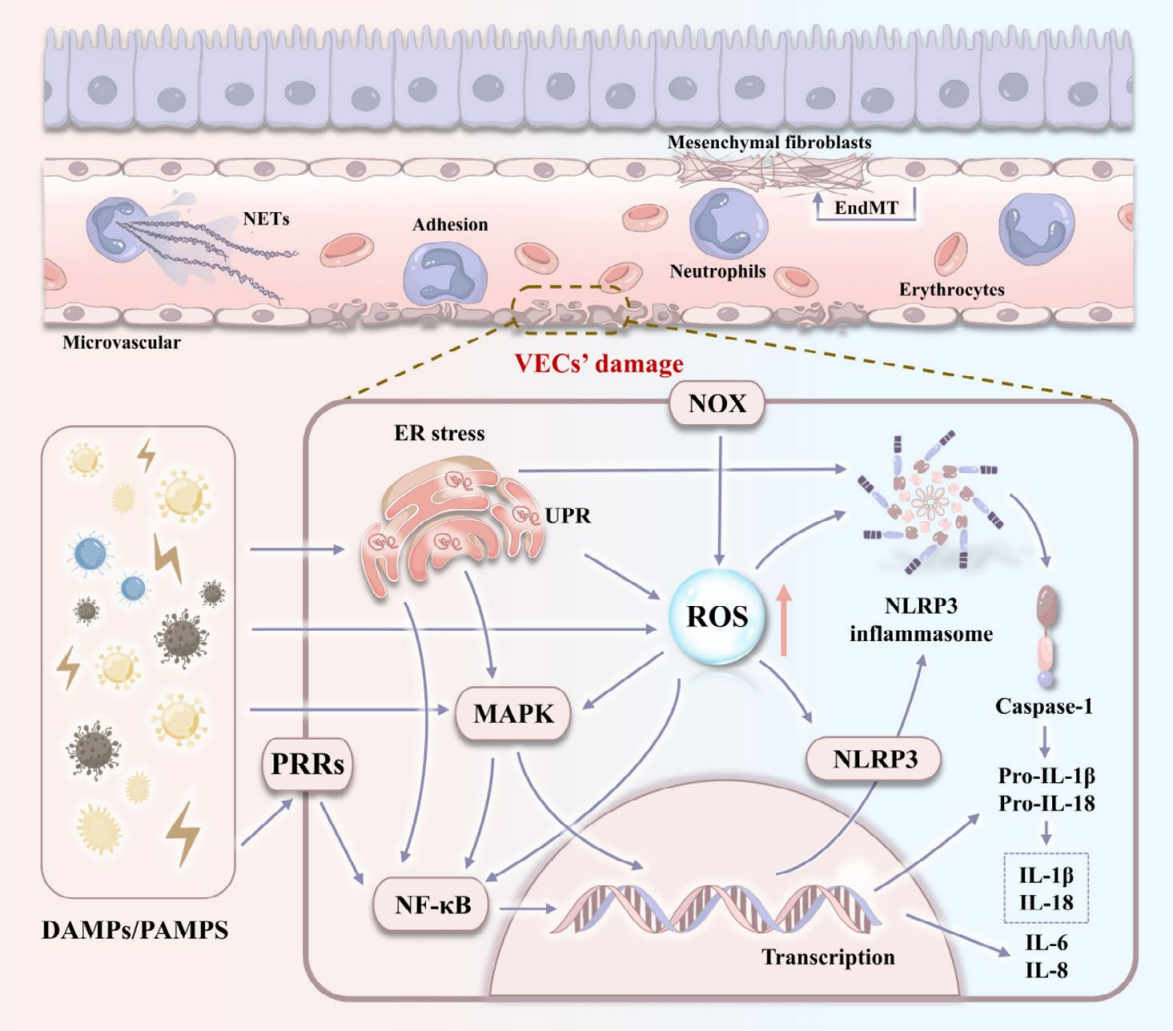
The mechanism of pulmonary VECs in the inflammation‐oxidation cycle. EndMT, endothelial‐mesenchymal transition; ER, endoplasmic reticulum; IL, interleukin; NETs, neutrophil extracellular traps; NLRP3, NLR family pyrin domain containing 3; NOX, NADPH oxidas; PRRs, pattern recognition receptors; ROS, reactive oxygen species; UPR, unfolded protein response.

In summary, inflammation and oxidative stress are both the body's defence mechanisms against external stimuli, and may also be key drivers of amplified tissue damage and abnormal remodelling. They form a dynamic regulatory network through mutual reinforcement to maintain the vicious cycle of pulmonary VECs injury. Figure 2 shows this complex cyclic reaction. Considering the important effects of inflammation and oxidative stress on the occurrence and progression of various lung diseases, this part is a promising site for intervention.

### 2.2 Genetic Factors

The genetic defects and epigenetic disorders of pulmonary VECs jointly constitute the genetic molecular basis for the development of lung diseases. The genetic variations in pulmonary VECs mainly exert their effects during disease progression through two distinct modes. One type of mutation directly induces disease and causes pulmonary VECs damage, while the other does not directly impair pulmonary VECs but indirectly increases the susceptibility of diseases by altering the key regulatory pathways or physiological processes. Both modes are clearly exemplified in the genetic variations associated with lung diseases [49, 50].

In hereditary PH, mutations in bone morphogenetic protein receptor 2 (BMPR2) in pulmonary VECs are present in approximately 75% of patients [51]. As a key receptor in the TGF‐β superfamily signalling pathway, BMPR2 mutations block SMAD 1/5/8 phosphorylation, leading to dysregulation of the expression of downstream target genes, such as the DNA binding protein inhibitor (ID‐1). This signalling disruption not only inhibits pulmonary VECs apoptosis, but also stimulates aberrant cell proliferation through the MAPK/ERK pathway, triggering EndMT, and further disrupting the vascular barrier [19]. In addition, deletion of the key transcription factor SMAD4 in the TGF‐β/BMP pathway exacerbates the signalling abnormalities, reduces the expression levels of target genes, and promotes the degradation of extracellular matrix through the activation of matrix metalloproteinases (MMPs), resulting in the deleterious cycle of proliferation‐fibrosis [52]. The potassium channel subfamily K member 3 (KCNK3) mutation is another genetic variant linked to PAH. This mutation disrupts energy metabolism in pulmonary VECs through metabolic reprogramming, and exacerbates cellular damage by impairing ion channel function [53]. Notably, Piper et al. found for the first time that RAB7 expression was reduced in pulmonary VECs of PAH patients, and knockdown of RAB7 resulted in the differential expression of 4842 genes, which radically altered the gene expression profile of pulmonary VECs, affecting mitochondrial metabolism and promoting cellular senescence [54]. This finding further suggests the lethal impact of core gene alterations on lung function and the cascading effect on downstream pathways.

At the level of DNA methylation regulation, abnormal DNA methylation mediated by methyltransferase 1 (DNMT1) could specifically target the promoter regions of antioxidant genes and directly inhibit their transcriptional activity [55]. This epigenetic modification leads to a significant downregulation of SOD and glutathione peroxidase (GPX) expression, impellers the cells' ability to inhibit ROS, and intensifies oxidative stress, thereby promoting cell damage [56, 57]. It emphasised how epigenetic diseases directly and significantly damage pulmonary VECs by interfering with the antioxidant system. It is worth noting that excessive accumulation of ROS within cells could inhibit the activities of DNA demethylase tet methylcytosine dioxygenase 2 (TET2) and silent information regulator 1 (SIRT1), leading to promoter hypomethylation or histone hyperacetylation [58, 59]. This, in turn, promotes the continuous high expression of inflammatory factors, further intensifying the inflammatory and oxidative reactions. This bidirectional interaction between oxidative stress and epigenetic abnormalities confirms their synergistic relationship. Similarly, the synergy and mutual reinforcement between epigenetic dysregulation and cellular senescence exacerbate the damage process of pulmonary VECs [60].

Beyond direct pathogenic effects, gene mutations can also increase disease susceptibility by disrupting the normal functions of pulmonary VECs. The expression of SRY box transcription factor 17 (SOX17), a pivotal regulator in maintaining the homeostasis of pulmonary VECs, is notably diminished by the common variants in its upstream enhancer region like rs10958403 and rs765727 [61, 62]. These variants disrupt the binding of HOXA 5/ROR‐α and affect the differentiation of alveolar capillary progenitor cells. It leads to abnormal synthesis of type IV collagen and elastin, affects the structural and functional integrity of the blood‐gas barrier, and increases the risk of interstitial lung disease (ILD) [50]. Studies on mice with endothelial‐specific SOX17 knockout had shown increased vascular permeability and inflammatory infiltration under hypoxic conditions [62]. Similarly, in individuals carrying functional variations of the protein convertase subtilisin/kexin type 9 (PCSK9) gene, the expression of very low‐density lipoprotein receptor (VLDLR) in lung tissue was upregulated several times. This upregulation increases viral cell binding, significantly raising the risk of acute exacerbation in patients with COPD [63]. However, the association research between pulmonary VECs gene abnormalities and the susceptibility to ARDS is still relatively limited. Cao et al.'s study on ARDS found that genetic factors such as TMEM176B, SLC2A5, CDC45, and VSIG8 might be associated with a high risk of disease [64], but the specific mechanisms by which these factors promote the occurrence and development of ARDS still need to be further explored.

The regulatory role of histone modifications in the senescence of pulmonary VECs has been increasingly recognised in recent studies. In an angiotensin II (Ang II) induced model of vascular endothelial cell senescence, the expression of the histone methyltransferase SET and MYND domain containing protein 2 (Smyd2) was significantly upregulated, and positively correlated with the senescence markers p21 and vascular cell adhesion molecule 1 (VCAM‐1), suggesting a potential involvement of Smyd2 in regulating the senescence phenotype [65]. Functional experiments further confirmed that Smyd2 overexpression can directly induce a typical senescence phenotype in pulmonary VECs, through the activation of intracellular senescence enhancers, disruption of normal cell cycle progression, and impairment of functional homeostasis, leading to decreased cell proliferation and secretory dysfunction [65]. Moreover, long‐term Smyd2 mediated histone modification abnormalities contribute to the development and progression of senescence‐associated lung diseases, highlighting the central role of epigenetic dysregulation at the histone modification levels in accelerating the senescence of pulmonary VECs, and promoting the pathogenesis of lung diseases [66].

In conclusion, epigenetic modifications exert multifaceted influences on pulmonary VECs' function, with their regulatory mechanisms displaying notable complexity. Aberrant modifications significantly impact pulmonary VECs' cellular homeostasis. Presently, research into pulmonary VECs' epigenetic regulation remains nascent, merely scratching the surface. Targeted epigenetic interventions hold promise for precise regulation of pulmonary VECs' function.

### 2.3 Cellular Senescence

**FIGURE 3:**
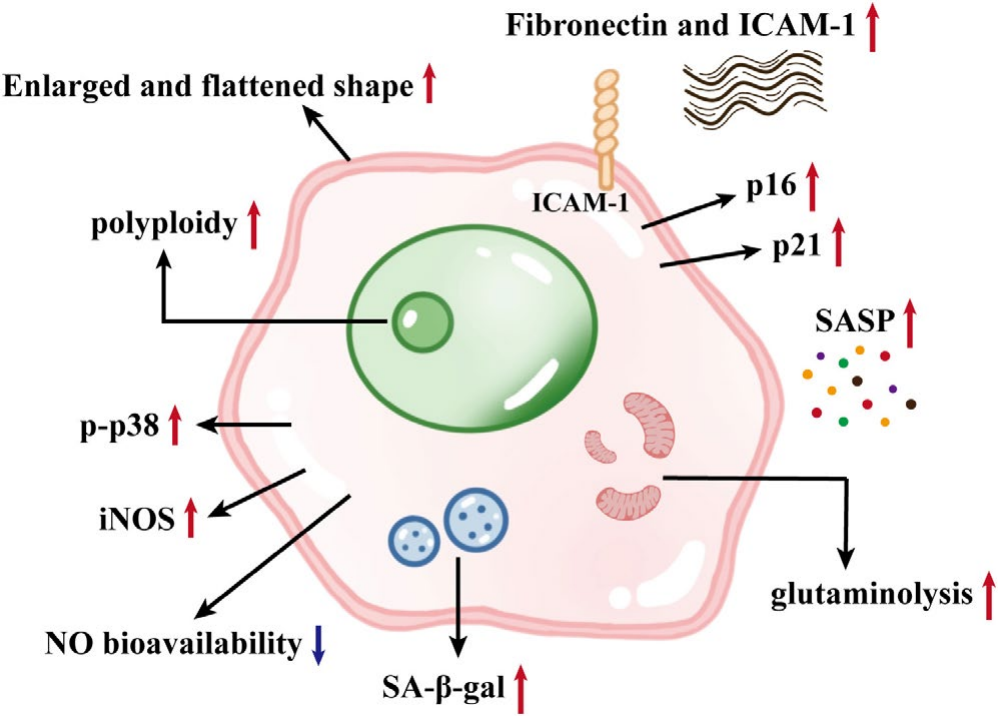
The main characteristics related to senescence of pulmonary VECs. NOS, nitric oxide synthase; ICAM‐1, intercellular cell adhesion molecule‐1; NO, nitric oxide; SASP, senescence‐associated secretory phenotype; SA‐β‐gal, senescence‐associated β‐galactosidase.

The aging process of pulmonary VECs shows dynamic change characteristics, and its core driving factors include continuous harmful stimuli and intrinsic metabolic abnormalities. Age‐related pulmonary VECs exhibit significant morphological and functional remodelling, including broad and flat morphological changes, upregulation of cyclin‐dependent kinase inhibitors such as p16/p21, accumulation of lysosomal age‐related β‐galactosidase (SA‐β‐GAL), and activation of the senescence‐associated secretory phenotype (SASP) [67]. Figure 3 shows the main characteristics of senescent cells. Clinical studies have shown that the expression levels of aging markers p16, p21, and SA‐β‐gal in lung tissues of patients with IPF and PAH were significantly increased, confirming that the accumulation of senescent cells was positively correlated with the severity of the diseases [68, 69]. Therefore, analysing the molecular mechanism of pulmonary VECs aging is a key entry point for achieving improvement or reversal of lung function.

Sustained DNA damage is a central driver of senescence in pulmonary VECs, which not only disrupts genomic integrity but also activates the ATM/ATR kinase signalling pathway, triggering a downstream cascade [70]. The activation of the damage repair system is accompanied by a significant upregulation of the p53‐p21 pathway, leading to irreversible cell cycle arrest in the G1/S phase and a typical senescence phenotype [71]. Systematic pooling of transcriptomics data from animal models of ALI demonstrated the enrichment of specific p53 and p21 dependent gene features and co‐occurrence of senescence markers, further confirming a strong link between this pathway and cellular senescence [72].

In addition, structural and functional alterations of telomeres are key components of pulmonary VECs aging at the genetic level that cannot be ignored. Under physiological conditions, telomere repeat sequences at the end of chromosomes gradually shorten with cell division due to inherent defects in the DNA replication mechanism [73]. When telomeres shorten to a critical length, the telomere uncapping event initiates a DNA damage response that activates the p53/p21 pathway and ultimately induces normal cellular senescence [74]. Telomere shortening can be accelerated by external stimuli. Cellular experiments have demonstrated that disturbed blood flow shortens telomere length in VEGs, and a similar phenotype has been observed in humans, where endothelial cells exposed to disturbed blood flow in vascular regions show significantly higher telomere attrition than in laminar flow regions [75, 76]. These findings suggest that the instability of the telomere shortening process is modulated by microenvironmental factors. Furthermore, blood flow disorders can induce pulmonary VECs senescence through the p53/p21 pathway in mice and cell models, providing indirect evidence suggesting that blood flow disorders accelerated telomere shortening [77]. However, the molecular mechanism of how blood flow disorders specifically disrupt the protective mechanism of telomeres remains to be explored.

The role of oxidative stress in the aging process of pulmonary VECs is particularly prominent, and radiation‐induced lung injury (RILI) is the most intuitive manifestation of oxidative reactions promoting aging. Excessive ROS not only directly oxidises the guanine‐rich repeat sequence in the telomere region, but also hinders telomere elongation by inhibiting the nuclear localisation of telomerase subunits [78]. Meanwhile, these active substances can also cause mitochondrial DNA (mtDNA) point mutations and DNA double‐strand breaks [79]. Analysis of autopsy samples from ARDS patients revealed a significant increase in aging‐related heterochromatin foci, confirming the promoting effect of inflammation–oxidative stress on the aging of pulmonary VECs [72]. It is worth emphasising that the interaction between aging and inflammatory oxidation reactions may not be unidirectional but rather mutually reinforcing and influencing each other. ROS can not only directly damage DNA, but also activate the NF‐κB signalling axis, initiating the transcription of inflammation‐related genes, and triggering the ‘inflammation‐oxidation’ cascade, in which SASP plays a key role [80, 81].

SASP is currently a hot topic in pulmonary VECs aging and is regarded as a key bridge between inflammation and aging. A large number of studies have confirmed that senescent cells can continuously secrete various inflammatory factors, chemokines, and MMPs, constructing a pro‐fibrotic and pro‐inflammatory microenvironment [82, 83, 84]. Furthermore, it can affect adjacent normal cells, induce DNA damage, activate a variety of signaling pathways, and promote originally healthy cells to enter the senescence state [75]. Finally, the factors secreted by SASP not only act on peripheral cells but also act on senescent pulmonary VECs themselves in an autocrine manner. The TGF‐β1 significantly upregulates the expression of EndMT markers [75, 76]. Studies have confirmed that SASP markers were significantly enriched in COPD, IPF, and PAH, suggesting their extensive association with the occurrence and development of lung diseases [85, 86, 87].

From the above, inflammation and oxidative stress can induce cellular senescence, and the SASP phenotype further aggravates local inflammation, forming a self‐perpetuating vicious cycle of ‘inflammation‐oxidative stress‐senescence’. Therefore, targeting and inhibiting the production of SASP or eliminating the secretion products related to SASP may become a potential strategy to break this cycle and improve the predicament of disease treatment.

### 2.4 Metabolic Abnormalities

#### 2.4.1 Metabolic Reprogramming

Metabolic reprogramming of pulmonary VECs is an important adaptive response to external stimulus signals or stress states, but its pathological transformation often becomes a key driving force for the progression of lung diseases. This metabolic remodelling is not a change in a single pathway, but involves the synergistic reconstruction of multiple pathways such as glycolysis, lipid metabolism, and amino acid metabolism [19, 88].

In terms of glucose metabolism, under hypoxic conditions, stably activated hypoxia inducible factor‐1 α (HIF‐1α) can preferentially upregulate the expression of glycolysis‐related enzymes such as hexokinase and phosphofructokinase, enabling cells to still rely on glycolysis for energy supply even after the partial pressure of oxygen is restored, thus forming the Warburg effect [89]. This similar effect also exists in COVID‐19‐related lung injury. Virus‐induced DRP1‐dependent mitochondrial fission can amplify the glycolytic reaction through the H₂O₂‐PHD2‐HIF‐1α axis, resulting in continued glycolytic activity even when the partial pressure of oxygen returns to normal, seriously hindering the repair process of endothelial function [90, 91]. Compared with oxidative phosphorylation, the ATP produced by glycolysis is only 1/18 of the former. This inefficient energy production model not only leads to a cellular energy crisis, but also the accumulation of lactic acid as a by‐product can cause intracellular acidification and damage the tight junction structure of the endothelial barrier [92]. In the hypoxia‐induced mouse pulmonary VECs model, the expression of key enzymes in glycolysis and the secretion of lactic acid were significantly increased, directly confirming the association between metabolic phenotype transformation and abnormal cell function [93]. The latest research shows that in hypoxic PAH models, endothelium‐specific enolase 1 (ENO1) could increase glycolytic flux by activating the PI3K/Akt/mTOR signalling axis, suggesting that multiple pathways could synergistically promote abnormal glycolysis [94]. In addition, single‐cell sequencing technology revealed that the glycolytic activity of pro‐inflammatory gCap subsets in senescent lung endothelium was abnormally elevated [95]. Senescent VECs exhibited abnormal activation of the glycolytic‐lactic acid axis, characterised by a significantly elevated expression of lactate dehydrogenase A (LDHA) compared to normal cells [96]. These studies suggest that metabolic reprogramming and cellular senescence form a cross‐regulatory network through energy metabolism pathways.

Lipid metabolism disorder is another important feature of metabolic reprogramming, and the endodermal lipid toxicity it causes plays a key role in the abnormal function of pulmonary VECs [97]. The excessive activation of PI3K/Akt signalling can inhibit the activity of AMPK [98]. On the one hand, it reduces the β‐oxidation process of fatty acids entering mitochondria. On the other hand, it promotes the expression of lipid synthesis related genes such as sterol regulatory element binding proteins (SREBPs). This leads to the accumulation of lipid components such as triglycerides, free fatty acids, and ceramides within pulmonary VECs. These lipid toxic substances can cause pulmonary VECs apoptosis and functional disorders by disrupting mitochondrial membrane integrity and activating ERS pathways [99]. Metabolomics analysis of patients with PAH revealed that the downregulation of mitochondrial carnitine palmitoyltransferase 1A (CPT1A) led to weakened fatty acid oxidation capacity and accumulation of lipotoxic compounds, further exacerbating endothelial damage [100].

It is worth noting that the disorders of glucose metabolism and lipid metabolism do not exist in isolation. Glycerol 3‐phosphate, an intermediate product of glycolysis, can serve as a precursor for lipid synthesis, forming a vicious cycle of sugar‐lipid metabolism [101]. This may be an important reason why metabolic reprogramming in lung diseases is difficult to reverse. In contrast, the role of amino acid metabolism in the metabolic remodelling of pulmonary VECs remains unclear, and its specific mechanism awaits further exploration.

#### 2.4.2 Mitochondrial Dysfunction

Mitochondria, as the energy factory of cells, their abnormal functions can directly trigger metabolic changes in pulmonary VECs and are the core driving factor for metabolic disorders in lung diseases. This abnormality is mainly manifested in multiple aspects such as mtDNA damage, imbalance in mitochondrial quality control, and disorder of the mitochondrial‐nuclear signalling pathway and forms complex interactions with mechanisms such as oxidative stress, inflammatory response, and aging.

As the core component of mitochondrial function, mtDNA damage is one of the initiation events of mitochondrial dysfunction. Unlike nuclear DNA, mtDNA lacks histone protection and has a limited repair mechanism, making it particularly sensitive to ROS [102, 103]. In lung diseases, oxidative stress is the most common inducer of mtDNA damage. High levels of ROS can directly attack the purine and pyrimidine bases of mtDNA, leading to the accumulation of oxidative damage products such as 8‐oxguanine and causing various forms of damage such as single strand breaks, point mutations, or fragment deletions [104]. These damages eventually lead to subunit synthesis disorders of the respiratory chain complex, reduce ATP production efficiency, force cells to shift to the glycolytic pathway to maintain energy supply, and form pathological associations with metabolic reprogramming. This type of injury was common in RILI and ARDS [105, 106].

Some studies have pointed out that in chemical lung injury models, after lung tissue is exposed to chlorine (Cl_2_), the level of 8‐oxoguanine DNA glycosylase 1 (OGG1) gradually decreases, leading to the accumulation of mtDNA oxidative damage and triggering ALI through the activation of pathways such as the NLRP3 inflammasome [107]. The findings of this study suggest a close connection between mtDNA damage and oxidative stress‐inflammatory responses. The related mechanism may be associated with the fact that damaged mtDNA fragments can be released into the cytoplasm or circulatory system as DAMPs. For instance, cytoplasmic mtDNA can initiate the NF‐κB pathway through Toll‐like receptor 9 (TLR9) recognition, or trigger type I interferon responses through the cyclic GMP‐AMP synthase (cGAS)‐interferon gene stimulating factor (STING) pathway, thereby causing systemic inflammation [108]. Clinical studies have shown that the mtDNA mutation rate of pulmonary VECs in patients with COPD was significantly increased, and the level of plasma mtDNA fragments was positively correlated with the concentrations of pro‐inflammatory factors such as IL‐6 and TNF‐α [109]. It is suggested that the release of mtDNA can form a persistent inflammatory microenvironment. The plasma mtDNA level of patients with severe COVID‐19 was positively correlated with the severity of the disease and the incidence of ARDS, further confirming the role of mtDNA damage in inflammatory amplification in the circulation [110]. This harmful cycle of oxidative stress‐mtDNA damage‐inflammation not only exacerbates mitochondrial dysfunction but also inhibits the mitochondrial repair mechanism through inflammatory signal feedback, accelerating the collapse of pulmonary VECs' energy metabolism. It also reveals the close connection between different mechanisms, which is also the common pathological basis of many lung diseases.

Abnormal mitochondrial quality control function is another key mechanism inducing mitochondrial dysfunction, mainly manifested as an imbalance of mitochondrial fission and fusion and dysfunction of mitochondrial autophagy. During the infection process of SARS‐CoV‐2, virus‐induced inflammatory factors can trigger dynamin‐related protein 1 (Drp1) phosphorylation by activating the RhoA‐ROCK signalling pathway, resulting in an increased degree of mitochondrial fragmentation [111]. The ROS and cytochrome released by fragmented mitochondria will further damage the integrity of the vascular endothelial barrier and aggravate pulmonary edema. In the hypoxia‐induced PH model, the continuous activation of Drp1 also disrupted the mitochondrial morphological balance, causing functional abnormalities in pulmonary VECs by altering their metabolic patterns and ultimately impairing the respiratory function of the lungs [112]. Furthermore, in hypoxic mouse models, after endothelial cells specifically knocked out the mitochondrial autophagy receptor FUNDC1, mitochondrial autophagy function was impaired, which in turn led to metabolic reprogramming and cellular senescence, ultimately promoting the occurrence and development of PAH [113]. Significantly, viruses such as SARS‐CoV‐2 could disrupt the mitochondrial autophagy process by targeting and inhibiting mitochondrial antiviral signalling proteins (MAVS), accelerating the damage and dissolution of pulmonary VECs [114].

These research results collectively highlight the significance of mitochondrial homeostasis in maintaining normal physiological functions of the lungs. They not only reveal the multi‐dimensional regulatory pathways that affect mitochondrial autophagy function and the severe pathological consequences they cause, but also provide an important theoretical basis for improving lung function by targeting and regulating mitochondrial quality control.

### 2.5 Immune Dysfunction

Pulmonary VECs, functioning as both guardians of the physical barrier and dynamic modulators of the immune microenvironment, play a crucial role in the complex immune network of the lung. Their involvement in immune regulation spans from innate immunity to adaptive immunity, and any disruption in this process can lead to severe immune‐related pathologies.

The surface of pulmonary VECs expresses a variety of PRRs, including Toll‐like receptors (TLRs), NOD‐like receptors, etc. [115]. Abnormal activation of PRRs can overly amplify the immune cascade reaction, induce local inflammatory storms, and ultimately lead to pulmonary VECs' damage. Studies have confirmed that in the SARS‐CoV‐2 infection model, the viral spike protein (S protein) can upregulate the expression of TLRs in pulmonary VECs through the angiotensin‐converting enzyme 2 (ACE2) receptor axis, triggering a severe inflammatory storm and causing more severe tissue damage [116]. Conversely, in the LPS‐induced ALI mouse model, blocking the TLR4 signal could effectively inhibit the activation of the NF‐κB pathway and the expression of IL‐6, significantly reducing the local inflammatory response [117]. These findings highlight the core role of PRRs in mediating local tissue damage. It is worth noting that the abnormal activation of PRRs can also form a vicious cycle with hypoxic stress. Under hypoxic conditions, the HIF‐1α pathway was cross‐activated with PRRs' signals, promoting pulmonary VECs to secrete chemokines such as CXCL8 and CCL2 and recruiting monocytes to migrate across the endothelium [118]. This process not only accelerates the destruction of the endothelial cell barrier but also further aggravates the local hypoxia state, forming an injury cycle.

Complement system is an important effector of innate immunity. Membrane attack complexes (MACs) generated by complement system activation can directly mediate cell lysis [119, 120]. Meanwhile, complement fragments can also act as pro‐inflammatory mediators, amplifying the inflammatory response triggered by PRRs and exacerbating tissue damage [121]. In a variety of lung diseases, the abnormal activation of complement system is a key factor mediating pulmonary VECs injury. Among severe COVID‐19 patients, lung biopsies showed that more than 90% of severe cases had pulmonary VECs injury, and the injury sites were accompanied by deposits of complement C5b‐9, C4d and so forth [122]. These complement components co‐localised with viral spike proteins, suggesting that the complement system continuously plays a mediating role in virus‐induced injury [122, 123]. In addition, the plasma C3a level in patients with post‐traumatic ARDS increased by two times, and was significantly positively correlated with mortality (*p* < 0.01) [123]. In patients with COPD, the expression of complement regulatory protein CD46 in lung tissue decreased by 30%, and was significantly correlated with the decline in the FEV1/FVC ratio (*p* < 0.001) [124]. These clinical data further confirm that complement activation is not only closely related to the severity of the disease, but also one of the core mechanisms mediating pulmonary VECs damage. It is worth noting that PRRs and the complement system do not act independently. The pro‐inflammatory factors released by the activation of PRRs can upregulate the expression of complement components, while complement fragments can in turn enhance the PRRs‐mediated signalling pathway. The two work together to form a damage amplifier at the innate immune level, dealing a fatal blow to pulmonary VECs. After the activation of the innate immune system, the communication between cells becomes crucial in determining the progression of the immune response.

Pulmonary VECs communicate with surrounding cells mainly through extracellular vesicles (EVs) [125], which play a significant role in both normal physiological conditions and pathological states. EVs are the core mediators for information exchange between pulmonary VECs and surrounding cells, connecting the regulatory networks of innate immunity and adaptive immunity [126, 127]. When lung tissue is in a pathological state, the secretion pattern of EVs becomes significantly abnormal. It no longer mediates physiological intercellular information exchange but becomes a disseminator of local pathological signals in lung tissue, continuously promoting tissue damage and remodelling, and has become a key driver to accelerate the progression of lung diseases. Among COVID‐19 patients, the level of blood EVs is positively correlated with the severity of the disease, and the bioactive molecules they carry are involved in pathological processes such as pro‐inflammatory responses, immune imbalance, and tissue damage and remodelling, confirming the key role of EVs in disease progression [128]. The pathological regulatory effect of EVs has been clearly confirmed in IPF and COPD. In IPF, EVs accelerate fibroblast activation and extracellular matrix deposition by delivering pro‐fibrotic factors, thereby promoting the fibrotic process [129]. In COPD, EVs can carry inflammatory mediators and proteases, exacerbating airway inflammation and lung tissue destruction, and are involved in emphysema and pulmonary artery remodelling [130, 131]. However, the heterogeneity of EVs is extremely high, with significant diversity in their types, sources, and the bioactive molecules they carry. Currently, the understanding of their subgroup classification and functional characteristics remains incomplete. Most existing studies focus on the role of a single EVs molecule or specific subgroups, lacking research on EVs systems for different diseases [132, 133]. Moreover, the synergistic or antagonistic relationships among different EVs subgroups and the network mechanisms by which they jointly participate in intercellular signal regulation have not been fully clarified. The resolution of these issues will provide a more precise theoretical basis for disease intervention targeting EVs.

Beyond the abnormalities in innate immunity and the pathological signal dissemination via EVs, dysregulation of the adaptive immune response also poses a significant threat to pulmonary VECs. Adaptive immunity is a more specific immune defence mechanism, and its dysregulation mediates pulmonary VECs injury mainly through targeted attacks mediated by autoantibodies and immune checkpoint molecules expression imbalance. Under abnormal autoimmune conditions, pulmonary VECs can become direct targets of adaptive immune responses. Autoantibodies form immune complexes by recognising specific antigens on the surface of pulmonary VECs, thereby activating the complement system and generating MACs [134, 135]. This will directly lead to the dissolution and necrosis of vascular endothelial cells, causing fatal damage to pulmonary VECs. The pathological mechanism of systemic sclerosis associated interstitial lung disease (SSc‐ILD) provides a typical example for this. Antinuclear antibodies in the patient's serum specifically recognise the surface antigens of pulmonary VECs, initiate complement cascade reactions, trigger complement dependent cytotoxicity (CDC) and antibody dependent cell mediated cytotoxicity (ADCC), and cause persistent endothelial injury [136].

The expression balance of immune checkpoint molecules on the surface of pulmonary VECs, such as programmed death ligand 1 (PD‐L1) and cytotoxic T lymphocyte associated protein 4 (CTLA‐4) ligands, is crucial for maintaining immune tolerance. When the expression of these molecules is downregulated, CD8^+^ T cells can become over‐activated and start to attack pulmonary VECs [137]. Analysis of lung tissues in patients with COVID‐19 and ARDS showed that PD‐L1 expression was significantly decreased, accompanied by increased levels of perforin and granzyme B, which in turn led to severe endothelial shedding and destruction of the alveolar capillary barrier [138, 139]. This clinical study provides direct evidence to confirm that the imbalance of immune checkpoints is an important inducement of pulmonary VECs damage. In addition, Wang et al.'s research found that the expression of PD‐L1 in lung tissue of patients with hypoxic PH is decreased and negatively correlated with the degree of pulmonary vascular remodelling [140]. It is suggested that it may be involved in the process of vascular remodelling, but its specific regulatory mechanism still needs further exploration. At present, research on immune checkpoint molecules mainly focuses on the field of oncology, while their tissue specific regulatory mechanisms in lung diseases remain unclear. Further studies are urgently needed to reveal their roles in maintaining pulmonary VECs function and disease progression.

**FIGURE 4:**
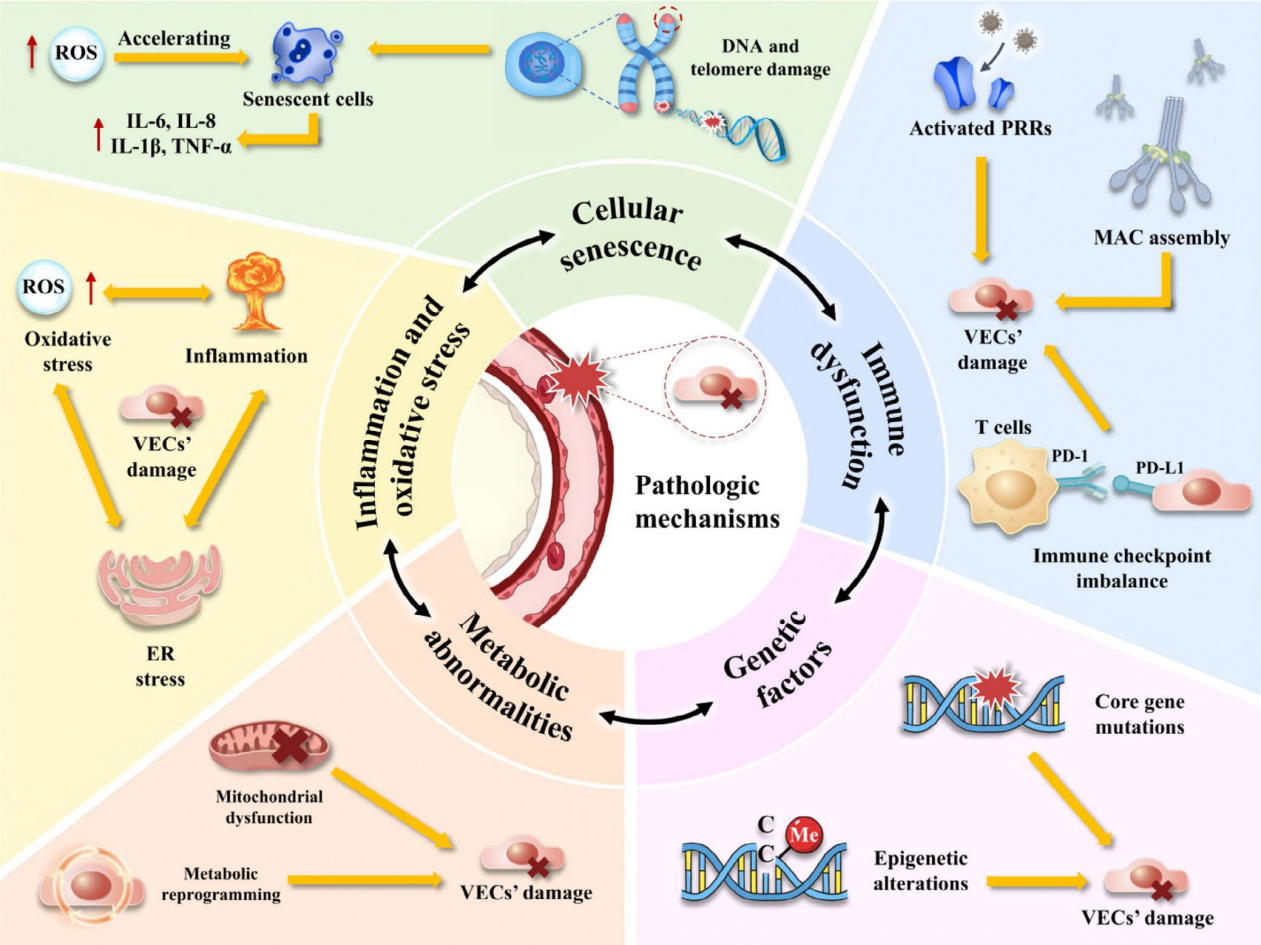
The main pathologic mechanisms of pulmonary VECs' injury. It is mediated by the interconnected mechanisms including inflammation and oxidative stress, cellular senescence, immune dysfunction, genetic factors, and mitochondrial abnormalities. These mechanisms synergistically amplify pulmonary VECs damage. DNA, deoxyribonucleic acid; ER stress, endoplasmic reticulum stress; MAC, membrane attack complex; PRRs, pattern recognition receptors; ROS, reactive oxygen species; SASP, senescence‐associated secretory phenotype; VECs, vascular endothelial cells.

Overall, the damage mechanism of pulmonary VECs in lung diseases constitutes a highly dynamic interactive network, with its core features reflected in the cascading feedback among multiple pathways such as inflammation, oxidative stress, genetic variation, cellular senescence, metabolic reprogramming, abnormal immune function, and mitochondrial dysfunction. Among them, inflammation, as the core driving hub, mediates tissue damage by initiating the inflammation‐oxidative stress cascade response, which not only destroys the cellular structure but also alters the cellular metabolic pattern, thereby accelerating the process of cellular senescence. Abnormal changes in cellular metabolism and the activation of SASP will in turn expand the inflammatory response, forming a vicious cycle of interaction. Genetic factors reshape the gene expression network through epigenetic modifications, alter the immune response threshold and cellular metabolic phenotype, significantly enhance disease susceptibility, and create favourable conditions for the occurrence of inflammation. Meanwhile, the pathological processes such as lung structure destruction, tissue remodelling, EndMT, endothelial lysis and tissue fibrosis caused by the interweaving of multiple mechanisms jointly constitute the pathological basis for the irreversible progression of diseases such as ALI/ARDS, PAH, IPF and COPD. Figure 4 presents the interaction framework between various pathological mechanisms and pulmonary VECs, which helps to understand the complex regulatory network of pulmonary VECs injury more intuitively.

## 3 Targeting VECs for Pulmonary Diseases

Pulmonary VECs, as the key regulators of pulmonary vascular homeostasis, their dysfunction is a common pathological hub driving the progression of various pulmonary diseases. Therefore, therapeutic strategies targeting pulmonary VECs as key targets have become an important breakthrough in the treatment of lung diseases. Table S1 summarises the current traditional therapeutic drugs targeting pulmonary VECs. However, due to the complexity and dynamic mutual feedback characteristics of the disease mechanism network, their therapeutic effect is often limited by a single target and the interaction of multiple pathways. Therefore, it is urgent to develop more targeted innovative treatment strategies starting from the core characteristics of the pathological mechanism of pulmonary VECs. The following text will systematically expound on the cutting‐edge treatment directions based on the above mechanisms, focusing on key pathological process nodes, and elaborate on the targeted innovative therapies and their transformation prospects.

### 3.1 Anti‐Inflammatory and Antioxidant Therapies

The inflammatory activation of pulmonary VECs involves multiple signalling pathways such as NF‐κB, MAPK, and NLRP3 inflammasome, and is closely related to the release of inflammatory factors [21, 141, 142]. Based on this, the core of anti‐inflammatory treatment lies in precisely blocking the abnormal activation of these pathways and eliminating inflammatory factors, thereby interrupting the cascade reaction. Dexamethasone (DEX), as a commonly used clinical drug, is precisely an effective anti‐inflammatory agent targeting this mechanism. It can induce the synthesis of Lipocortin‐1, and inhibit the activity of phospholipase A₂, thereby blocking the NF‐κB pathway in cells and reducing the synthesis and release of cytokines such as TNF‐α, IL‐1, and IL‐6 [143, 144]. Studies have confirmed that in COVID‐19 related ARDS, the 28‐day mortality rate of mechanically ventilated patients treated with dexamethasone (6 mg/d × 10 days) was reduced by 33%, fully demonstrating its clinical value [145]. However, due to the special physiological structure of pulmonary capillaries and factors such as the first‐pass effect, the drug concentration in pulmonary capillaries is often limited. Coupled with the influence of systemic side effects, the therapeutic application of dexamethasone is greatly restricted. The emergence of new targeted drug delivery systems (DDSs) has brought hope for solving these problems [146].

**FIGURE 5:**
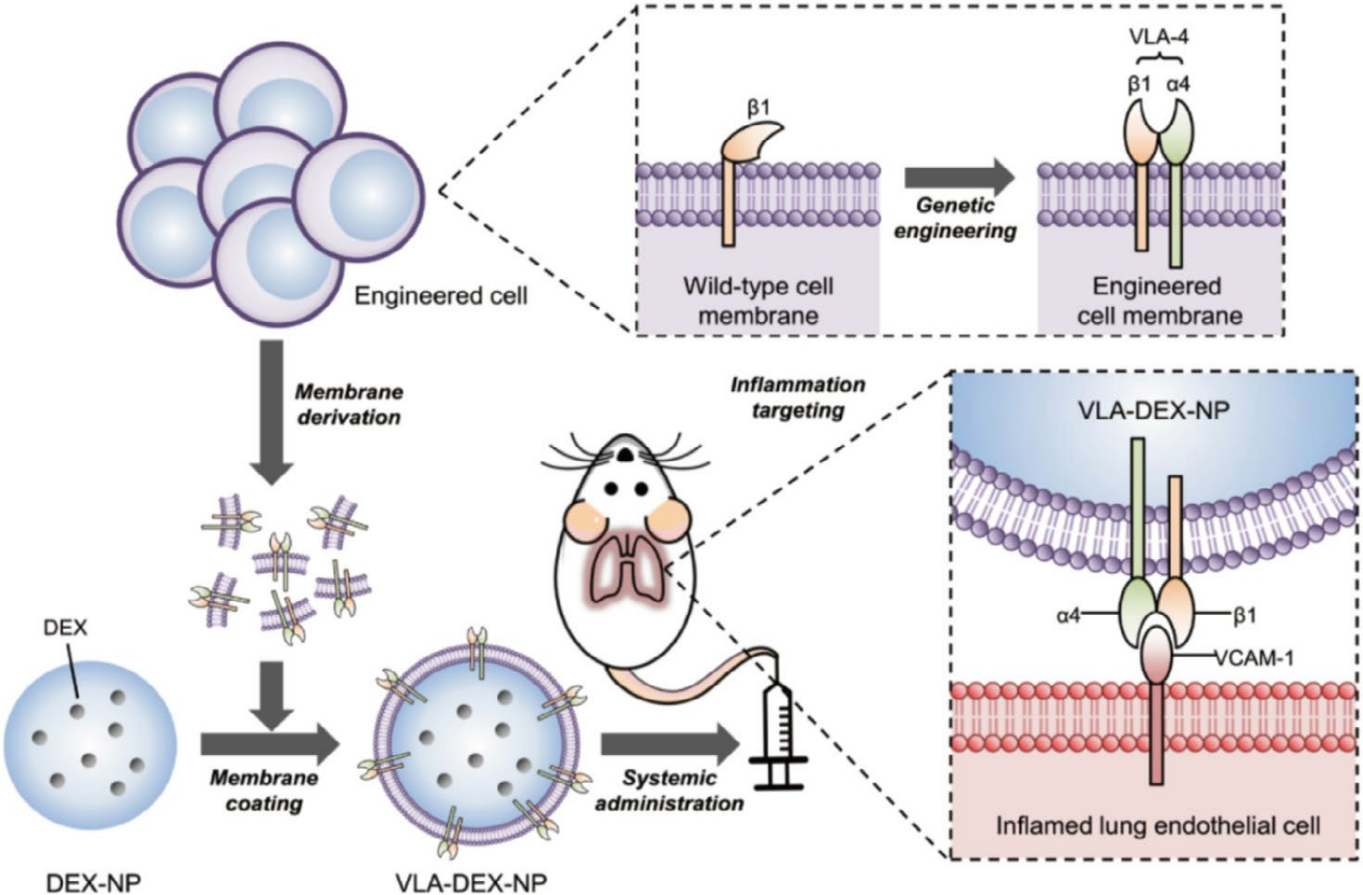
Schematic illustration of genetically engineered cell membrane coated nanoparticles for targeted drug delivery to pulmonary VECs. Wild‐type cells were genetically engineered to express VLA‐4, which is composed of integrins α4 and β1. Then, the plasma membrane from the genetically engineered cells was collected and coated onto dexamethasone‐loaded nanoparticle cores (DEX‐NP). The resulting VLA‐4 expressing cell membrane coated DEX‐NP (VLA‐DEX‐NP) can target VCAM‐1 on inflamed lung endothelial cells for enhanced drug delivery. Reprinted from Park et al., 2021 with permission [157].

DDSs is an advanced technology that precisely delivers drugs to target tissues or cells using specific carriers [147, 148]. These vectors can achieve targeted delivery based on the characteristics of pulmonary VECs. According to the targeting mechanism of the vectors, they can be classified into three major categories: receptor‐mediated vectors, antibody‐conjugated vectors, and vectors with special physicochemical properties [149, 150, 151, 152, 153, 154, 155]. Table S2 summarises the DDSs designed for the characteristics of pulmonary VECs. Nayak et al. developed dendritic nanoparticle carriers targeting antibodies against aminopeptidase P2 on the surface of pulmonary VECs. SPECT–CT imaging and biodistribution studies had shown that after intravenous injection of this nanomaterial, the uptake in the lungs of rats reached 45% within 5 min, while in the control group it was only 1.1%, with the targeting efficiency increased by more than 40 times [156]. This case fully demonstrated the powerful carrying capacity of DDSs. With the assistance of DDSs, the anti‐inflammatory efficacy of DEX has been comprehensively enhanced. In recent studies, nanoparticles coated with genetically engineered cell membranes (VLA‐NP) had been used to target and deliver DEX to pulmonary VECs [157]. The cell membranes of these nanoparticles expressed VLA‐4, which could specifically bind to VCAM‐1 highly expressed on pulmonary VECs. Figure 5 shows the preparation process and principle of this specific material. Compared with the traditional intravenous injection of DEX, the accumulation of VLA‐NP in the lungs of inflamed mice increased by 2.5 times. Furthermore, free DEX led to a 40% increase in plasma creatinine levels, while VLA‐DEX‐NP maintained a safe creatinine level and had no nephrotoxicity. This novel targeted method effectively alleviated the systemic toxicity associated with free DEX, demonstrating high efficacy and safety [157]. Another innovative nanomaterial (ML/LA@DEX NPs) was composed of a myeloid cell membrane wrapped with DEX liposomes, bringing a revolutionary breakthrough to the treatment of inflammation. This material activated CD11b through the leukocyte adhesion molecular agonist (LA‐1), enhancing its binding ability to inflammatory endothelial cells ICAM‐1, and simultaneously competitively occupied the adhesion sites of neutrophils, significantly improving the targeting efficiency. The accumulation of this material in the lungs of ALI mice was 2.6 times higher than that of ordinary liposomes [158]. It confirmed that DDSs had outstanding advantages of high efficiency, safety, and strong specificity in the treatment of lung diseases, and had broad application prospects. A nanomaterial (AB‐INRplex), which used rod‐like indomethacin nanocrystals (INRs) as the carrier to load SOD and modify anti‐ICAM‐1 antibody (Ab) through electrostatic interaction, showed strong antioxidant function [159]. In the LPS‐induced mouse model, the uptake of Ab‐INRplex in the lungs was 40% higher than that of free SOD, and the ROS level was 2.8 times lower than that of the control group. Meanwhile, thanks to the synergistic effect of indomethacin, this material had a strong anti‐inflammatory effect. The levels of TNF‐α, IL‐6, and COX‐2 in it are respectively 3 times, 2.5 times, and 1.5 times lower than those in the control group. This characteristic of simultaneously inhibiting the storm of inflammatory factors and oxidative stress can effectively break the vicious cycle of inflammation‐oxidative damage [159].

In addition, the discovery of endothelial progenitor cells (EPCs) provides a new strategy for targeted anti‐inflammatory and antioxidant therapy of pulmonary VECs. Studies have confirmed that EPCs are one of the stem cell subtypes with the strongest anti‐inflammatory and antioxidant effects, and showed outstanding anti‐inflammatory and antioxidant capabilities in the intervention of lung diseases [160, 161]. In the local pulmonary inflammatory microenvironment, EPCs can achieve all‐round intervention through multi‐dimensional synergistic effects of reducing endothelial damage, repairing endothelial function, anti‐inflammation, and anti‐oxidation, highlighting significant therapeutic potential. Specifically, in terms of anti‐inflammatory effects, EPCs can significantly reduce the release of pro‐inflammatory factors such as TNF‐α and IL‐6 by releasing nitric oxide (NO) and inhibiting the NF‐κB signalling pathway, thereby alleviating inflammatory damage to pulmonary VECs [160]. Meanwhile, EPCs can highly express core antioxidant enzymes such as SOD and GPX, which can directly eliminate excessive ROS in the lungs and effectively alleviate oxidative stress damage. In addition, unlike other stem cells, EPCs also have the potential to differentiate directionally into mature pulmonary VECs [160]. In the area of pulmonary vascular injury, they can precisely migrate and directly integrate into the damaged endothelial layer. They can not only efficiently repair the damaged vascular barrier but also promote the formation of new blood vessels by secreting key angiogenic factors such as vascular endothelial growth factor (VEGF) and angiopoietin‐2 (Ang‐2) [160, 162, 163]. Based on these advantages, treatment options for EPCs are constantly emerging, and EPC transplantation is a feasible treatment strategy [164]. Mao et al. transplanted EPCs into an LPS‐induced ALI rat model. The assessment after 7 days showed that compared with the control group, lung injury was reduced by approximately 45%. This confirmed the powerful anti‐inflammatory ability of EPCs [165]. However, this method has significant limitations. EPCs may accumulate unpredictable gene mutations during in vitro amplification, and there is a risk of abnormal proliferation and tumour formation after transplantation [166]. In particular, some mutations (such as TP53 mutations) may be below the conventional detection threshold, and microenvironmental factors may further exacerbate the abnormal behaviour of these mutant cells [167]. In addition, such therapies also face serious ethical and long‐term safety issues. The above limitations all restrict the clinical transformation of EPC transplantation technology and require further in‐depth research.

Compared with the EPCs transplantation strategy, the EPCs derived extracellular vesicles (EPC‐EVs) show more significant application advantages and safety [168, 169]. Studies have found that EPC‐EVs could exert therapeutic effects by transporting anti‐inflammatory miRNAs, such as functional molecules like miR‐126 and miR‐103‐3p [170, 171]. They could reduce the generation and release of inflammatory mediators in lung tissue by specifically inhibiting inflammation‐related signalling pathways such as NF‐κB and phosphatidylinositol 3‐kinase/protein kinase B (PI3K/Akt). This could block the activation of multiple inflammatory cascade reactions, disrupt the inflammatory amplification cycle, and achieve precise regulation of lung inflammation [172]. The research of Zhou et al. further confirmed that EPC‐EVs could replace complete EPCs for the treatment of sepsis‐associated pneumonia, effectively avoiding the technical problems faced by stem cell transplantation, such as immune rejection and low cell survival efficiency [173]. Specifically, EPC‐EVs delivered miR‐126‐3p and miR‐126‐5p to target cells, precisely targeting and inhibiting the expression of key inflammatory regulatory factors such as high mobility group protein B1 (HMGB1) and VCAM‐1, ultimately significantly improving pulmonary microvascular dysfunction [173]. In the sepsis mouse model, after intravenous injection of exosomes, the survival rate was significantly increased (52% vs. 13%, *p* < 0.05), pulmonary edema was significantly improved (wet/dry ratio 4.0 vs. 5.2, *p* < 0.05), and liver and kidney function damage was also alleviated [173]. However, at present, there are still key scientific issues that urgently need to be explored in the EPC‐EVs scheme. For example, whether the single EPC‐EVs program can achieve the same effect as the direct transplantation of EPCs in the core therapeutic effects of anti‐inflammatory, anti‐oxidation, and promoting vascular repair. The differences between the two in terms of action intensity, duration, and mechanism still need to be further clarified through comparative studies. In addition, in areas such as the applicability of EPC‐EVs in other types of lung diseases, in vivo pharmacokinetic characteristics, and systematic preclinical safety evaluation, more high‐quality studies are also needed to provide evidence support.

### 3.2 Gene Therapies

With the in‐depth analysis of genetic regulatory mechanisms in the pathogenic network of pulmonary VECs, gene therapy has become an important strategy to break through the limitations of traditional treatments. Based on the molecular basis of the abnormal function of pulmonary VECs, the current research mainly focuses on the delivery of functional genes to compensate for pathogenic gene defects and the precise regulation of epigenetic diseases to realise the intervention of pulmonary VECs. Investigating these aspects is crucial for accurately reversing the pathology of pulmonary VECs, repairing pulmonary vascular function, and establishing new avenues for targeted treatments of lung disease.

Gene delivery technology provides an effective means to compensate for abnormal mutations in core genes [174]. Take the mutation or downregulation of BMPR2, a key causative factor of PAH, as an example. Recombinant adenovirus (AAV) mediated gene delivery has demonstrated significant efficacy. Reynolds et al. pioneered the use of an AAV vector (AdCMVBMPR2) to deliver the human BMPR2‐myc fusion gene to pulmonary VECs [175]. The strategy effectively restored lung tissue BMPR2 expression to near normal levels in a model of wild larkspur (MCT) induced hypoxic PH. This was accompanied by a decrease in TGF‐β secretion and suppression of the EndMT process, further confirming that this strategy was effective in reversing pulmonary vascular remodelling [175]. On this basis, the development of a new generation of AAV vectors has further expanded the application scenarios. For example, AAV1 mediated SIN3a gene delivery could reverse the hypermethylation of the BMPR2 promoter and restore its expression through epigenetic regulation [176]. Although AAV therapies have significantly improved pulmonary hemodynamic parameters in animal models, their clinical translation still needs to overcome the core issues of immunogenicity, targeted delivery efficiency, and long‐term safety. Non‐viral vector delivery systems offer a promising alternative to circumvent the inherent limitations of viral vectors. The lipid nanoparticles (LNP) developed by Cao et al. could be efficiently loaded with BMPR2 mRNA, and after being taken up by pulmonary VECs via endocytosis, the mRNA could be efficiently released and its functional expression could be realised [177]. Not only was the system significantly less immunogenic, it was also 1/10 the cost of adenoviral therapy and had good potential for clinical translation. In the MCT model, the 0.1 mg/kg dose group restored the phosphorylation level of SMAD1/5/9 to 85% of that in the normal group. However, LNP mRNA therapy currently requires frequent intravenous injections to maintain efficacy, and its durability is inferior to that of AAV [177]. In addition, although data based on a rat model showed that the efficiency of pulmonary endothelial transfection was greater than 75%, differences in the composition of human plasma proteins may significantly affect the targeting efficiency and pharmacokinetic properties of LNP, and its clinical applicability requires further validation.

Epigenetic modulation is not only an important dimension of gene therapy, but also an intervention program with broad applicability, which can play a key regulatory role in regulating cell growth and development, altering cellular metabolism, modulating inflammatory phenotypic shifts, and other pathological processes [178, 179, 180]. Among them, the CRISPR/dCas9 system enables epigenetic regulation of lung diseases through precise gene editing. Studies have shown that fusing dCas9 with DNA methyltransferase DNMT3A and targeting the promoter region of pro‐fibrotic genes could silence their expression by increasing DNA methylation [181]. In a mouse model of PF, it reduced collagen deposition in lung tissue by 40%, and significantly attenuated the pathological changes of fibrosis. Despite challenges like low delivery efficiency and off‐target risks, this approach effectively reduced fibrosis. It is worth mentioning that Chen's team developed a stable CRISPR‐Cas9 ribonucleoprotein LNP delivery system, achieving a breakthrough of 16% lung editing efficiency in a mouse intravenous injection model [182]. This progress has significantly enhanced the practical application of the CRISPR/dCas9 system.

Significantly, epigenetic modulators targeting DNA methylation, histone modification, and non‐coding RNA abnormalities have also been widely used in experimental studies. For example, the use of DNMT1 inhibitors reversed aberrant methylation of key genes to restore SOD2 expression and reduced ROS accumulation, thereby alleviating pulmonary VECs damage [183]. Tian et al. found a 60% decrease in DNMT1 expression and a 40% decrease in the area of PF following the use of a DNMT1 inhibitor in MCT‐induced PAH rats [184]. Of interest, exploration of the mechanism revealed that DNA aberrant methylation could alter the cellular metabolism of pulmonary VECs, which provided strong evidence for a close association between epigenetics and metabolism. Besides, histone deacetylase (HDAC) inhibitors have also been widely explored in the field of histone modification regulation. Applegate et al.'s team applied vorinostat to a newborn calf model of hypoxic PH and found a significant decrease in HDAC1 protein expression in the treated group [185]. Although it did not reduce pulmonary artery pressure, it improved hypoxia‐induced right ventricular dysfunction and reduced vascular stiffness. Furthermore, miRNA mimic therapies hold promise for epigenetic regulation of pulmonary VECs. Yan et al. developed liposomal antisense miR‐21 (LP‐anti‐miR‐21) to competitively inhibit miR‐21, thereby suppressing SMAD7 protein, enhancing TGF‐β signalling, and reducing PF [186]. In a mouse PF model, LP‐anti‐miR‐21 reduced miR‐21 levels in the lung by 40.2% and decreased FN1 and COL1A1 protein expression by 69.1% and 66.7%, respectively, significantly improving the Ashcroft pathology score. Despite these results, the complex network of non‐coding RNAs suggests that targeting a single miRNA or lncRNA may activate compensatory pathways, potentially diminishing therapeutic effects and warranting further investigation.

### 3.3 Anti‐Aging Therapies

Senescence is a central mechanism in the complex pathogenesis of pulmonary VECs injury. Multiple factors, including oxidative stress, DNA damage, and aberrant epigenetic modifications, synergistically drive pulmonary VECs senescence. Senescent pulmonary VECs initiate SASP, continuously releasing pro‐inflammatory mediators, chemokines, and MMPs. This remodels the local inflammatory microenvironment, recruiting more inflammatory cells and directly contributing to abnormal vascular wall remodelling and excessive extracellular matrix deposition, which are key pathological features in diseases such as IPF, PAH, and COPD. Effective anti‐aging therapies must target the core mechanisms. This requires protecting genome integrity and promoting DNA damage repair to delay senescence initiation, as well as removing senescent cells and inhibiting SASP release. Ultimately, the detrimental cycle of damage–aging–re‐damage must be broken to restore pulmonary VECs homeostasis.

Telomere attrition, an irreversible marker of senescence in pulmonary VECs, has emerged as a central target for interventions aimed at mitigating the aging process [187, 188]. The primary strategies involve maintaining telomere length through telomerase activation, and directly safeguarding telomeres and nuclear DNA from damage, both of which have shown promise in various experimental models [189, 190]. In terms of telomerase activation, telomerase reverse transcriptase (TERT) activators are the research focus. Astragalus extract, as the most widely studied TERT activator, has a significant effect in improving age‐related pulmonary lesions by upregulating TERT expression and prolonging telomeres [191]. A new type of small molecule TERT activator (TAC) offers a potential solution. It upregulates TERT transcription by directly activating the MEK/ERK/AP‐1 pathway and induces DNMT3B‐mediated methylation of the p16 promoter, achieving dual effects of telomere protection and senescence inhibition [192]. Experimental data show that long‐term treatment with 0.5 μM TAC can increase telomere length to 1.8 times that of the control group (*p* < 0.001), and reduce the number of DNA damage foci (TIFs) induced by telomere dysfunction by 60%. The density of senescent cells in the tissues of aged mice decreased by 50%, providing a possibility for long‐term intervention of chronic lung diseases. And EPAS1, a key telomere protection factor in cells that is also expressed in human VECs [192]. It had confirmed that EPAS1 agonist (M1001) significantly upregulated the expression of the human telomere protective proteins (TRF1 and TRF2) and the DNA repair factor (RAD50). In a bleomycin‐induced animal model, treatment with M1001 resulted in an approximately 50% reduction in TIFs, and a 30% decrease in the proportion of cells positive for the senescence marker SA‐β‐gal [192]. The application of antioxidants is also an effective way to achieve telomere protection and senescence inhibition by reducing oxidative damage. It was found that Mentha piperita extract could effectively reduce ROS production [193]. In the H₂O₂ stress model, 0.25 mg/mL of the extract reduced the protein carbonylation level from about 200 fluorescence signalling units (RFUs) to 50 RFUs. And the long‐term treatment (6 weeks) reduced the telomere shortening rate from 150 to 100 bp (*p* = 0.0091), which significantly delayed senescence. In terms of vasoprotection, the extract reduced vascular adhesion molecule (VCAM) expression (*p* = 0.0289) and ROS levels (*p* = 0.0079), and improved vascular permeability (RFU from 40 to 20, *p* < 0.0001) [193]. Notably, antioxidants have been shown to inhibit pulmonary VECs senescence by suppressing the core regulatory pathway of the SASP.

Eliminating accumulated senescent pulmonary VECs is a direct means to block their pathological effects, and senolytics as a targeted therapy for eliminating senescent cells show broad prospects. Among them, the combination of dasatinib and quercetin was currently the most thoroughly studied protocol [194]. The mechanism of action of this plan lay in eliminating senescent cells, reducing the release of related inflammatory factors and proteases, and inhibiting the occurrence and development of the disease. In an exploratory study on the application of this protocol in COPD, the results of its in vitro experiments showed that this combination could reduce the expression of the aging marker p16INK4a in cells by 50% [195]. In the mouse model exposed to smoke, the level of p21WAF1 protein in lung tissue decreased significantly, while the total number of inflammatory cells, neutrophils, and macrophages all decreased. Furthermore, recent studies have found that intermittent supplementation of flavonoids could improve vascular endothelial function and arterial stiffness in aged mice by selectively eliminating senescent cells [196]. Flow cytometry results confirmed that fisetin could reduce the number of p16^+^ cells by 85%, and had no significant effect on normal endothelial cells and smooth muscle cells, demonstrating its targeting of senescent cells. Cellular experiments further confirmed selectivity. Regarding senescence markers, 1 μM fisetin reduced SA‐β‐gal activity by 50% (*p* < 0.05) and p16 gene expression in human aortic endothelial cells (HAECs) by 71% (*p* < 0.001). It also decreased collagen I by 44% (*p* = 0.070) and advanced glycosylation end products (AGEs) by 36% (*p* = 0.030) [196]. It should be noted that senescent endothelial cells can inhibit excessive proliferation through paracrine signalling. However, the uncontrolled clearance of these cells may lead to unregulated vascular remodelling and capillary loss, underscoring the critical importance of the timing of clearance [77, 197]. Early clearance may hinder capillary regeneration, while delayed intervention could improve fibrosis. Therefore, targeted clearance has become the core direction of subsequent treatment development.

### 3.4 Metabolic Abnormality Intervention

The key to intervening in abnormal cellular metabolism lies in reversing pathological metabolic reprogramming and restoring normal cellular energy metabolism patterns on the one hand, and improving mitochondrial dysfunction and re‐establishing cellular energy homeostasis on the other. These two directions together constitute the core strategy of metabolism‐targeted therapy for lung diseases.

Metabolic reprogramming is a hallmark of pulmonary VECs in pathological conditions. Specifically, aberrant upregulation of glycolysis and dysregulation of fatty acid metabolism are the predominant metabolic alterations observed. Targeting the key molecular players governing these metabolic pathways has emerged as a promising therapeutic strategy [93, 198]. Aberrant activation of 6‐phosphofructo‐2‐kinase/fructose‐2, 6‐bisphosphatase 3 (PFKFB3), a pivotal regulatory enzyme in the glycolytic pathway, serves as a crucial marker of metabolic reprogramming in pulmonary VECs [199]. The PFKFB3 inhibitor, 3‐(3‐pyridinyl)‐1‐(4‐pyridinyl)‐2‐propen‐1‐one (3PO), effectively targets this pathway, reducing glycolytic flux and thereby suppressing aberrant angiogenesis [200]. A notable advantage of 3PO was its transient inhibition of the glycolytic branch pathway without significantly affecting the basal metabolism of normal tissues, thus minimising systemic toxicity. Carnitine palmitoyltransferase 1A (CPT1A) is crucial in lipid metabolism as the rate‐limiting enzyme in fatty acid β‐oxidation (FAO) [201]. Etomoxir inhibits CPT1A, preventing fatty acids from entering mitochondria for β‐oxidation and decreasing acetyl coenzyme A production [202]. The use of etomoxir in the LPS‐induced ALI mouse model resulted in significant improvements in energy metabolism and lung injury. Specifically, ATP levels were approximately 0.5 nmol/mg in the ALI group, while they rebounded to approximately 1.0 nmol/mg in the CPT1A‐inhibited group (*p* < 0.05) [203]. These findings highlight the potential of targeting metabolic processes to address abnormalities in pulmonary VECs.

Direct damage to mitochondria, the cellular energy factories, results in an immediate collapse of pulmonary VECs' metabolism [204]. Remodelling endothelial metabolic homeostasis through mitochondrial supplementation has emerged as a promising strategy. The development of mitochondrial transplantation technology facilitates this goal [205]. Therapies now exist for direct or systemic supplementation with autologous derived mitochondria, which can replace dysfunctional mitochondria, enhance cellular bioenergetics, and reduce oxidative stress, thereby supporting the application of mitochondrial transplantation [206, 207]. In the field of lung disease, the team of Cloer et al. applied the technology to ameliorate ischemia–reperfusion injury (IRI) in transplanted lungs. In a porcine model, IRI‐injured lungs were treated with a significant decrease in pulmonary vascular resistance (PVR), an increase in oxygenation capacity, a 65% decrease in the necrosis marker pMLKL, a decrease in the expression of the apoptosis marker PARP, and a 25% increase in cell survival [208]. Interestingly, Lin et al. delivered in vitro isolated mesenchymal stromal cells (MSCs) mitochondria directly to pulmonary VECs by mimicking the active transfer of functional mitochondria from MSCs to damaged endothelial cells by means of microtubule nanotubes (TNTs) [209]. TNTs‐mediated transfer was only 25% efficient, whereas artificial transplantation was able to achieve a 90% efficiency, which resulted in the enhancement of metabolism of pulmonary VECs [209].

Enhancing cellular metabolism by modulating quality control mechanisms, such as mitochondrial fragmentation, fusion, and autophagy, is a crucial approach to mitigating metabolic dysfunction in pulmonary VECs. This strategy has been extensively investigated across various lung disease models [210, 211]. Under pathological conditions, Drp1 interacts with Fis1, leading to mitochondrial disruption and cell death [212]. The P110 peptide, an inhibitor of the Drp1‐Fis1 interaction, effectively blocks this process and has demonstrated efficacy in several lung disease models [213]. A research team developed SC9, a small molecule inhibitor that restored Drp1 mitochondrial association signalling to near baseline levels in LPS‐treated cells [214]. In a LPS‐induced mouse model, SC9 reduced the respiratory distress score from 2.9 to 0.4 (an 86% decrease) and significantly lowered plasma inflammatory factors. Future validation is anticipated for its efficacy in COPD, ARDS, and other lung diseases. In addition, in terms of regulation of mitochondrial fusion, MFN2 and OPA1 are key proteins that regulate mitochondrial outer and inner membrane fusion, respectively [215, 216]. Li et al. demonstrated that in a model of cigarette smoke (CS)‐induced COPD, the use of leflunomide and BGP15 significantly enhanced the expression of MFN2 and OPA1, promoted mitochondrial fusion, reduced mitochondrial fragmentation, and decreased intracellular and mitochondrial reactive oxygen species levels, thereby restoring cellular function [217]. For mitochondrial autophagy, eliminating damaged mitochondria is vital. Urolithin A (UA) has been shown to enhance mitochondrial autophagy via the PINK1/TAX1BP1 pathway, significantly increasing autophagosome‐mediated sequestration of damaged mitochondria [218]. In a CuO NPs‐induced injury model, UA reduced ROS levels by 60% and decreased cell death from 35% to 15%, absent in PINK1 knockout cells. In vivo studies in mice demonstrated that UA improved the clearance of damaged mitochondria and lowered oxidative stress markers in vascular tissues [218].

The above indicates that reshaping metabolic homeostasis and restoring mitochondrial dysfunction are essential in disrupting the metabolism–mitochondrial damage–oxidative stress cycle. Thus, therapeutic strategies for pulmonary VECs have evolved from focusing on metabolic pathways to comprehensive mitochondrial function regulation, playing a crucial role in mitigating cellular senescence and interrupting the oxidative stress cycle.

### 3.5 Immunomodulatory Therapies

For immunomodulatory therapies, they contain innate immunity and adaptive immunity. Any activation in this process can identify and eliminate antigenic foreign substances, maintain the physiological dynamic balance and relatively stable physiological functions, and achieve effective therapies of lung diseases.

Hyperactivation of PRRs triggers pulmonary VECs to initiate an innate immune response and provoke an inflammatory storm. Among these receptors, the Toll‐like receptor 4 (TLR4) signalling pathway is pivotal [219]. Extensive research focuses on interventions targeting this pathway, leading to the development of several drugs that mitigate the pathological processes of various lung diseases by acting at different pathway sites. The chalcone‐derived compounds 7w and 7x, developed by Zhang et al. as novel myeloid differentiation 2 (MD2) inhibitors, specifically blocked the binding of MD2 to TLR4, inhibited the formation of the TLR4/MD2 complex, and significantly attenuated lung tissue injury in ALI mice [220]. Ruscogenin (RUS), the main active ingredient of the traditional Chinese medicine maitake, had also been shown to reduce immune activation of pulmonary VECs and alleviated particulate matter (PM) induced ALI by interfering with TLR4/MyD88 signalling [221]. Additionally, a pyrithione‐rich extract (PEC) bound to both TLR4 and MD2, and reduced LPS‐induced lung tissue damage by inhibiting the interaction between the two [222]. In the field of PF, Dou et al. found that ginseng and eustoma extracts could reduce extracellular matrix (ECM) deposition and ameliorate PF by inhibiting the TLR4 pathway [223]. Radicicol (SFN) had also shown potential in the treatment of COPD by targeting the TLR4 signalling pathway [224]. Effective inhibition of the TLR4 pathway, a key node in the development of innate immunity and inflammation, is a promising therapeutic strategy that can significantly ameliorate the symptoms of various inflammation‐associated lung diseases.

It is worth mentioning that in the field of targeted intervention for the complement system, inhibiting the complement system to reduce the production of MACs and thereby lower their direct damage to pulmonary VECs is a highly promising and feasible approach at present [225, 226]. In the context of COVID‐19 intensive care, the application of anti‐C5A monoclonal antibody (Vilobelimab) has shown remarkable effects, reducing the 28‐day mortality rate of patients receiving mechanical ventilation by 23.9% [227]. More recently, the novel complement C3 inhibitor CP40‐KK, developed by Dai et al., resulted in a 36% reduction in vascular thickening and a 70% decrease in plasma C3a levels in an MCT induced rat model of PAH, significantly improving the disease phenotype [228]. In lung IRI, complement fragments also had a synergistic effect with neutrophil infiltration and oxygen free radical release, further aggravating lung tissue damage [229, 230]. Based on the above, the future development direction of complement targeted therapy should consider integrating multi‐disciplinary technical means to build a multi‐target intervention system.

**FIGURE 6:**
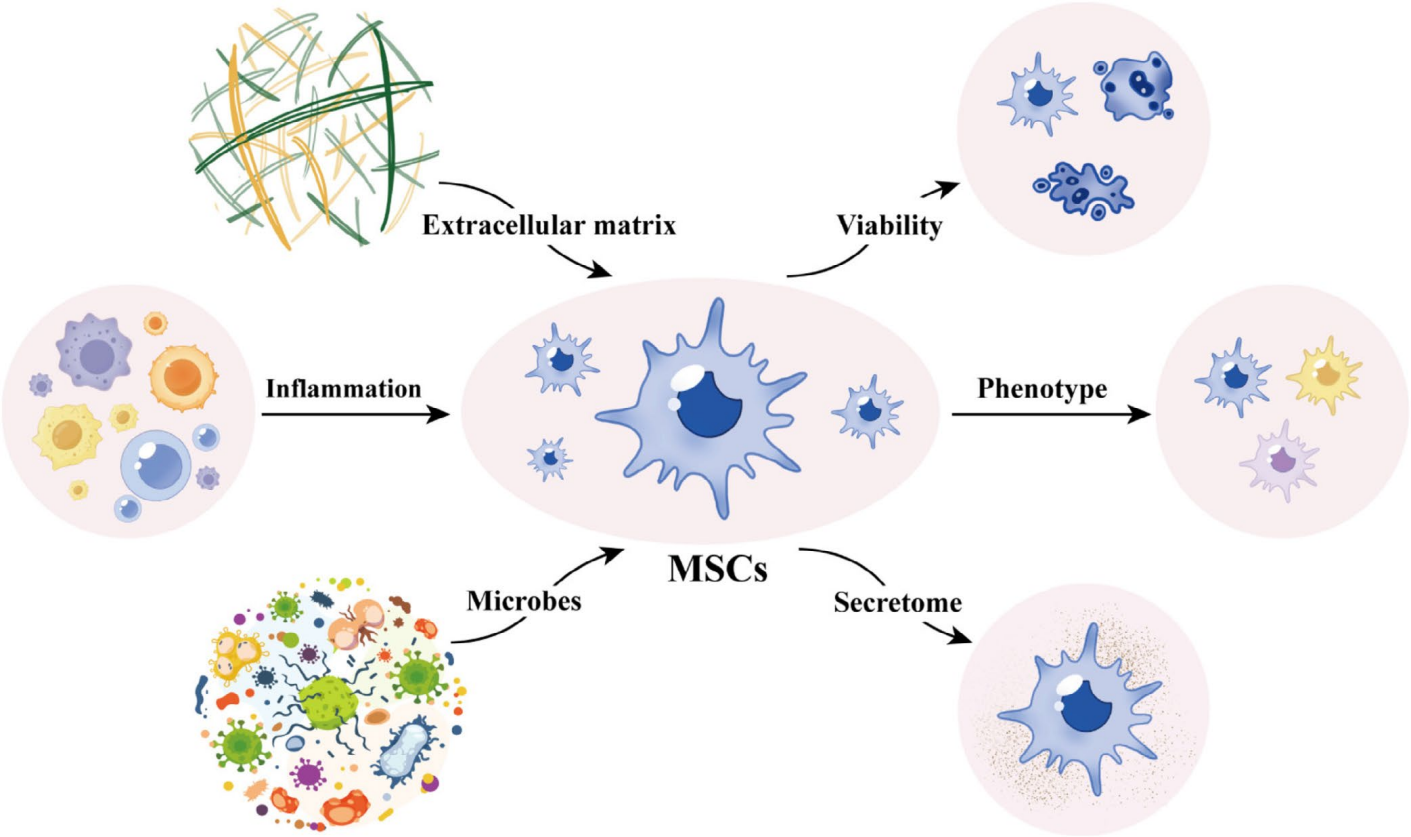
The core characteristics of MSCs. The microbiome, inflammation, and extracellular matrix are shown as external factors that directly influence and activate MSCs. In response to these signals, MSCs modulate their core biological programs: survival and health status (viability), differentiation potential and cellular identity (phenotype), and bioactive factors (secretome). Through these coordinated outputs, MSCs ultimately execute their functions in immunomodulation, tissue repair, and homeostasis maintenance. MSCs, mesenchymal stem cells.

MSCs are currently one of the most widely used types of stem cells in the research of pulmonary VECs therapy [231, 232]. Figure 6 illustrates the different functional properties that MSCs can present in response to different stimuli. Compared with EPCs, although it is weaker in functions such as anti‐inflammation and vascular repair, it has systematic immunomodulatory capabilities [233]. MSCs can precisely regulate the activation and function of various immune cells, such as T cells, B cells, and macrophages, through direct contact or paracrine pathways, thereby suppressing excessive immune responses from the source [231]. It is worth noting that the unique in vivo biological distribution characteristics of MSCs further amplify their application value in the treatment of lung diseases [234]. After intravenous administration, a significant pulmonary first‐pass effect occurs, with approximately 50% to 80% of the cells remaining in the pulmonary capillary network during the initial circulation stage [235]. This characteristic enables it to act more directly on the immune disorder areas of the lungs. Multiple animal experiments have confirmed that intravenous injection of MSCs can significantly improve the tissue damage of pulmonary vascular endothelial cells in the ALI mouse model. One of the core mechanisms is to reduce the damage to the endothelium through efficient immune regulation [236, 237]. However, due to the significant differences in the secretion profiles of MSCs from different sources, the consistency of therapeutic effects is affected. Intravenous infusion may cause transient fever or hemodynamic fluctuations, and its response to different injury microenvironments varies greatly, which restricts the stability of the therapeutic effect [238, 239, 240, 241]. The MSC‐EVs therapy derived from this has become a research hotspot because it retains the immunomodulatory characteristics of MSCs and avoids some risks of cell transplantation [242]. In preclinical models, MSC‐EVs have demonstrated the ability to improve vascular barrier function, regulate vascular permeability, and maintain immune homeostasis, making them promising for the treatment of ARDS, COPD, and PF [243, 244, 245]. Interestingly, the therapeutic efficacy of MSC‐EVs can be optimised through specific pretreatment strategies. For instance, Rozier et al. reported that stimulating MSCs with interferon‐gamma (IFN‐γ) led to changes in the composition of the secreted EVs, enhancing their immunomodulatory and antifibrotic properties, and significantly ameliorating PF in a mouse model of systemic sclerosis [246]. Furthermore, the transfection of MSCs with target protein encoding plasmids via lentiviral vectors has enabled the construction of specifically targeted EVs, further enhancing their therapeutic potential [247]. However, the complexity of the preparation process and the inherent biological instability of MSC‐EVs have posed challenges to their widespread practical application.

Finally, modulation of the PD‐1/PD‐L1 axis and correction of its pathological imbalance may mitigate the damage to pulmonary VECs caused by aberrant immune activation, thereby preserving lung function [140]. Notably, the directionality of intervention regarding this pathway must be dynamically adjusted according to the specific disease microenvironment. In the PAH model, hypoxia caused the downregulation of PD‐L1 expression in pulmonary VECs and attenuation of PD‐1/PD‐L1 signalling, which triggered Th17 cell differentiation and immune‐mediated injury [140]. In this context, supplementation of PD‐L1 protein or induction of endothelial‐specific PD‐L1 overexpression via an adeno‐associated virus vector could restore pathway activity, inhibit Th17 differentiation, and attenuate pulmonary vascular remodelling [140]. In contrast, in the PF model, abnormally high PD‐L1 expression on the cell surface hijacked immunosuppressive signals, contributing to T cell depletion and collagen deposition. Blockade of the PD‐1/PD‐L1 axis (e.g., anti‐PD‐L1 monoclonal antibody) reversed the over‐suppression, restored T cell function, and attenuated fibrosis, as demonstrated in a mouse model of PF [248]. These strategies, although intervening in opposite directions, all aim to restore immune homeostasis by targeting pathological deviations in the PD‐1/PD‐L1 pathway. While the regulation of this pathway is well established in cancer immunotherapy, its application in lung diseases remains in the exploratory stage.

**FIGURE 7:**
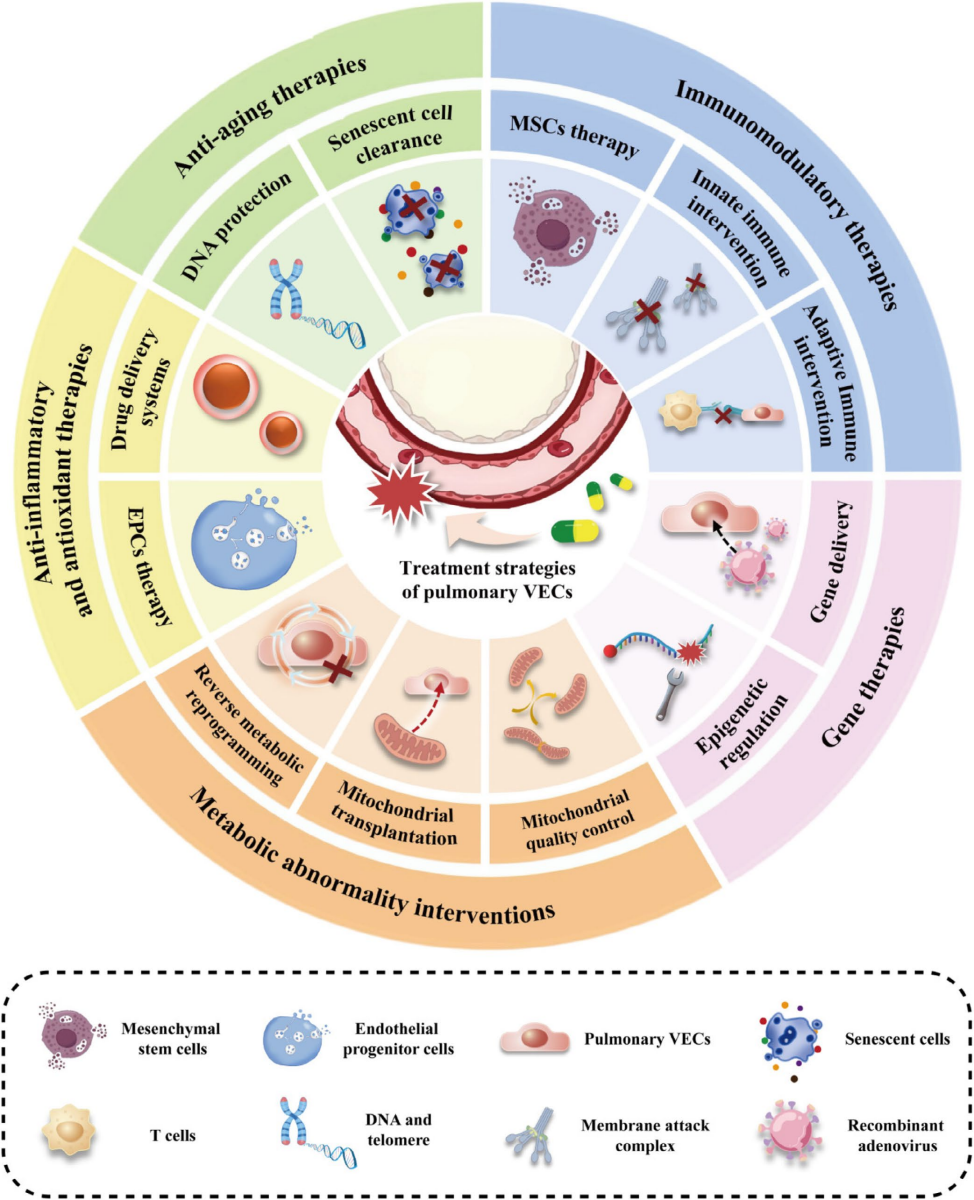
The novel pulmonary VECs targeted therapeutic strategy for lung‐related diseases.

From the perspective of pulmonary VECs, targeted intervention studies have been conducted based on their specific pathogenic mechanisms in different lung diseases, including ALI, ARDS, COPD, PAH and PF. As shown in Figure 7, this figure presents a comprehensive intervention framework for pulmonary VECs dysfunction. From the dimensions of anti‐inflammatory and antioxidant therapies, gene therapies, anti‐aging strategies, metabolic abnormality intervention, and immunotherapy, combined with innovative technologies such as drug delivery technology, gene editing tools, and cell therapy, the precise regulation of pulmonary VECs function has been initially achieved. However, these intervention strategies still face several common challenges in the process of clinical translation, and these challenges have also clarified the exploration direction for subsequent research.

## 4 Perspectives

The development of therapeutic strategies targeting pulmonary VECs has seen significant progress, yet clinical translation remains a critical challenge in this field. The primary obstacle lies in optimising precise delivery systems. The unique hemodynamic characteristics of the pulmonary vasculature and the heterogeneity of pulmonary VECs make it difficult for existing carriers to be uniformly distributed at the lesion site, resulting in substantial variations in therapeutic efficacy [249, 250]. While various lung‐targeted nanocarriers have been developed and their targeting capabilities demonstrated in animal models, these carriers faced significant hurdles in long‐term safety assessment, stability in complex in vivo environments, as well as scale‐up and standardised preparation [251, 252]. Existing vectors generally suffer from difficulties in scale‐up preparation, limited generalisability, low carrier compatibility, and insufficient adaptation to the dynamic lung microenvironment [253]. Therefore, the development of new generation delivery systems with high stability, strong generalisability, good safety, and a simple manufacturing process is essential to streamline the drug development process and accelerate the clinical translation of targeted therapies.

The limitations of animal models pose a significant challenge in translating research findings to clinical applications. Current lung disease research heavily relies on rodent (mice and rats) models, but their pathological processes often fail to fully replicate the complexity and chronicity that characterise human lung disease, resulting in limited clinical predictive value. Experimental models currently available cannot accurately simulate the heterogeneity of endothelial cells observed in human lung disease [254, 255]. Single cell studies have revealed the existence of distinct endothelial subpopulations in the human lung that responded differentially to injury signals, a nuance that was difficult to capture in standard animal models [35]. Additionally, key physiological differences between rodents and humans, such as the lack of fine bronchioles and the more complex airway branching in the human lung, make it challenging to accurately simulate small airway lesions and assess their impact on respiratory function [256, 257]. Furthermore, critical physiological parameters like lung volume and airflow rate differ quantitatively between animals and humans. Finally, multifactorial etiologies, including genetic susceptibility, chronic environmental exposures, and infections, often underlie human lung diseases [258, 259]. For instance, commonly used mouse models of LPS or acid aspiration only simulated a single inflammatory pathway, failing to capture the full complexity of the disease [260]. Optimising and developing animal models more relevant to human diseases is crucial for bridging the gap between basic research and clinical applications. Future models should integrate multiple factors, such as combined exposure or gene editing with environmental stimuli, to better simulate pathological complexity. Large animals such as pigs and sheep are more similar to humans in terms of the distribution of bronchial glands, the anatomical structure of lung lobes, and the hemodynamic characteristics of the heart and lungs [261, 262]. However, while enhancing modelling strategies to improve translational medicine's predictive value, caution is necessary to avoid over‐extrapolating conclusions due to interspecies differences.

The limited efficacy of novel therapeutic strategies is hindered by multiple factors, posing a significant challenge for clinical translation. Current therapeutic research often focuses on a single target or mechanism, which is prone to compensatory pathway activation, making it difficult to achieve ideal and durable therapeutic efficacy [263, 264]. Therefore, synergistic intervention strategies are a necessary direction to overcome the limitations of current single target therapies. In this review, it reveals the complexity of the network of mechanisms associated with pulmonary VECs injury and the significant cascade feedback effects among pathways. Based on this, the multi‐mechanism and multi‐strategy synergistic interventions are expected to significantly improve the therapeutic effects for pulmonary VECs related diseases. For instance, the combined use of anti‐inflammatory drugs could block the initiation of the inflammation‐oxidative stress cascade [141]. At the same time, senescent cell scavengers and telomere protectants were applied to reduce the load of senescent cells and the release of SASP, thereby breaking the vicious cycle of damage‐senescence‐inflammation [265]. On this basis, metabolic regulators were used in combination to repair the metabolic function of pulmonary VECs, maintaining their energy homeostasis and physiological state [266, 267]. It was supplemented with immunomodulators to alleviate immune‐mediated pulmonary VECs injury, ultimately achieving all‐round protection of the endothelial barrier. The proposed therapeutic approach dynamically adapts to the disease progression. During the acute phase, the focus is on rapidly mitigating the initial damage through anti‐inflammatory, antioxidant, and immunomodulatory interventions. In the chronic phase, the strategy emphasises anti‐aging, metabolic regulation, and the inhibition of vascular remodelling and fibrosis. For genetically driven diseases, the integration of gene repair or downstream pathway modulation is also considered. This synergistic approach covering the core mechanisms of inflammation initiation‐aging amplification‐metabolic collapse‐immune abnormality is expected to achieve better therapeutic efficacy.

In the future, the exploration of new targets and the innovation of cutting‐edge technologies will further break through the limitations of current treatments. The dynamic changes in epigenetic modifications of pulmonary VECs, their complex intercellular communication networks, and their perception and response mechanisms to mechanical forces such as blood flow shear force are all expected to become highly promising novel therapeutic targets. With the in‐depth application of artificial intelligence (AI) in target mining, drug design and efficacy prediction, as well as the continuous breakthroughs of synthetic biology technology in the development of engineered cell therapy vectors, the research and development process of new small molecule drugs and biologics will be significantly accelerated [268, 269]. The novel nucleases such as xCas9 and SpRY developed by protein engineering have broken through the PAM sequence limitations of the traditional CRISPR system, greatly expanding the target range of gene editing and providing more powerful tools for precisely repairing pathogenic genes or regulating epigenetic states [182, 270]. Meanwhile, the new generation of cell therapies represented by chimeric antigen receptor T cell (CAR‐T) technology, by engineering T cells to specifically target and eliminate pulmonary VECs or related pathogenic cells under pathological conditions, also offers promising possibilities for breakthrough treatments of lung diseases [271, 272].

## 5 Conclusions

Dysfunction of pulmonary VECs, as central regulators of pulmonary circulation homeostasis, contributes to lung diseases like ALI/ARDS, PH, PF, and COPD through a cycle of inflammatory‐oxidative stress, genetic and epigenetic abnormalities, cellular senescence, metabolic disruptions and immune disorders. Innovative therapeutic strategies targeting pulmonary VECs have shown promise in repairing the endothelial barrier, reversing vascular remodelling and enhancing treatment efficacy. Future efforts should focus on clinical translation, promote synergistic multi‐mechanism interventions, and integrate AI and single‐cell multi‐omics technologies to shift from symptom management to molecular precision interventions in the treatment of lung diseases.

## Author Contributions

Q.L., H.Z., and L.L. were responsible for the conception and design of the manuscript. Q.L. and M.G. prepared and wrote the manuscript. J.L. collected resources. L.L. and F.L. revised the manuscript. All authors contributed to the article and approved the final revision of the manuscript.

## Conflicts of Interest

The authors declare no conflicts of interest.

## Supporting information


**Table S1:** Traditional therapeutic drugs for pulmonary VECs targeting therapy.

**Table S2:** The novel drug delivery systems targeting pulmonary VECs.

## Data Availability

The data that support the findings of this study are available from the corresponding author upon reasonable request.
