# Supplementary material for: Pulmonary Vascular Endothelial Cells in Lung Diseases: Mechanisms, Therapeutic Strategies, and Future Directions
Source: Cell Prolif. 2025 Oct 15;59(2):e70136. doi: 10.1111/cpr.70136 (PMC12877960; doi:10.1111/cpr.70136)
Supplement: Supplementary file 1 — Table S1: Traditional therapeutic drugs for pulmonary VECs targeting therapy. [file CPR-59-e70136-s002.docx]

Supplementary Table 1. Traditional therapeutic drugs for pulmonary VECs targeting therapy

| Classification | Drug | Mechanism | Disease | Reference |
| --- | --- | --- | --- | --- |
| Statins | Atorvastatin, Simvastatin, Ulinstatin | Anti-inflammatory, antioxidant, anti-thrombotic, lipid regulator. | COPD, ILD | [6] |
| Enzymes | Glutathione | Antioxidant, scavenging free radicals, promoting proliferation and repair. | ARDS, COPD | [7] |
| NSAIDs | Aspirin | Inhibits micro thrombosis and improves endothelial  Function. | COPD, PE | [8] |
| ACEI/ARB | Captopril, Irbesartan | Improve pulmonary circulation and reduce pulmonary edema. | COPD, ALI | [9] |
| Phosphodiesterase inhibitors | Dipyridamole, Cilostazol | Inhibits local thrombosis, improves local oxygen supply, and reduces injury. | COPD, PH, PE | [10] |
| Vasodilators | Sodium nitroprusside nitroglycerin | Relaxation of vascular smooth muscle, resulting in vasodilation. | ARDS, COPD | [11] |
| GCs | Prednisone, Dexamethasone | Reduces inflammatory response and endothelial cell damage. | ARDS, COPD, ILD | [14] |
| Neutrophil elastase  inhibitors | Sivelestat sodium | Inhibition of neutrophil elastase activity. | ARDS, ALI | [14] |

GCs, Glucocorticoid; COPD, Chronic obstructive pulmonary disease; ILD, Interstitial lung disease; PH, Pulmonary hypertension; PE, Pulmonary embolism; ARDS, Acute respiratory distress syndrome; NSAIDs, Non-steroidal anti-inflammatory drug; ACEI, Angiotensin-converting enzyme inhibitors; ALI, Acute lung injury; ARB, Angiotensin receptor blocker.
