# Supplementary material for: Pulmonary Vascular Endothelial Cells in Lung Diseases: Mechanisms, Therapeutic Strategies, and Future Directions
Source: Cell Prolif. 2025 Oct 15;59(2):e70136. doi: 10.1111/cpr.70136 (PMC12877960; doi:10.1111/cpr.70136)
Supplement: Supplementary file 2 — Table S2: The novel drug delivery systems targeting pulmonary VECs. [file CPR-59-e70136-s001.docx]

Supplementary Table 2. The novel drug delivery systems targeting pulmonary VECs

| **Carrier type** | **Carrier** | **Mechanism** | **Receptor** | **Receptor characteristic** | **Reference** |
| --- | --- | --- | --- | --- | --- |
| Receptor mediated carriers | LA NPs | Low molecular weight heparin fragment on the carrier surface specifically binds to P-selectin. | P-selectin | Pre-stored, fast response, short duration, expressed by both platelets and endothelial cells, weak specificity. | [149] |
|  | DIP-FU-PPy NPs | FU sulfate part and P-selectin specific binding. | P-selectin | Pre-stored, fast response, short duration, expressed by both platelets and endothelial cells, weak specificity. | [150] |
|  | V-Nar/ICG/LN | Synthetic peptide sequence of special and VCAM 1 specific binding. | VCAM-1 | Synthesis after stimulation of inflammatory factors. Distributed in VECs and hematopoietic cells. | [151] |
|  | SA-C-N NPs | SA has a high affinity for E-selectin. | E-selectin | Delayed synthesis, expressed only in endothelial cells, high specificity and expression persistence. | [152] |
|  | ML/LA-1@DEX NPs | The binding of CD11b to ICAM-1 is expressed on the myeloid cell membrane coated on the surface of the vector. | ICAM-1 | Widely distributed, delayed expression, extensive functions. | [158] |
| Antibody-coupled carrier | PECAM@BM NLCs | Anti-PECAM-1 antibody and receptor specificity. | PECAM-1 | High expression at resting state cell junctions. | [153] |
|  | VCAM/MLT NLCs​​ | Anti-VCAM-1 antibody and receptor specificity. | VCAM-1 | inflammatory factors. Distributed in VECs and hematopoietic cells. | [154] |
|  | ICAM-NLC/Pro/Ang | Anti-ICAM-1 antibody and receptor specificity. | ICAM-1 | Widely distributed, delayed expression, extensive functions. | [155] |
| Special property carrier | iGeoCas9 RNP-LNPs | Cationic lipid particles carry positive charges and combine with the negative charges on the surface of VECs through electrostatic adsorption. | Not applicable | | [182] |

NPs, Nanoparticles; LA-1, Leukocyte adhesin-1; BM, Bardoxolone methyl; LA, LMWH+ASTA; LMWH, Low molecular weight heparin; ASTA, Astaxanthin; DIP, Dipyridamole; FU, Fucoidan; PPy, Polypyrrole; SA, Sialic acid; Dex, Dexamethasone; ML, Myeloid cell membrane-chimeric liposomes; NLCs, Nanostructured lipid carriers; MLT, Melatonin; Ab, Antibodies; INR, Indomethacin nanorods; LNPs, Lipid nanoparticles; PPC, Plasma protein corona; Nar, Naringenin-loaded; ICG, Indocyanine green-labeled; LN, Lipid nanoemulsions; VLA-4, Very late antigen-4; Pro, protamine; Ang, angiopoietin-1; RNP, Ribonucleoprotein; ICAM-1, Intercellular cell adhesion molecule-1; PECAM-1, Platelet endothelial cell adhesion molecule-1; VCAM-1, Vascular cell adhesion molecule-1; VECs, Vascular endothelial cells.
